# Genomic epidemiology of CVA10 in Guangdong, China, 2013–2021

**DOI:** 10.1186/s12985-024-02389-9

**Published:** 2024-05-30

**Authors:** Huimin Lian, Lina Yi, Ming Qiu, Baisheng Li, Limei Sun, Huiling Zeng, Biao Zeng, Fen Yang, Haiyi Yang, Mingda Yang, Chunyan Xie, Lin Qu, Huifang Lin, Pengwei Hu, Shaojian Xu, Hanri Zeng, Jing Lu

**Affiliations:** 1 School of Public Health, Southern Medical University, Guangzhou, China; 2 Guangdong Provincial Institution of Public Health, Guangdong Provincial Center for Disease Control and Prevention, Guangzhou, China; 3 Guangdong Provincial Key Laboratory of Pathogen Detection for Emerging Infectious Disease Response, Guangdong Workstation for Emerging Infectious Disease Control and Prevention, Guangdong Provincial Center for Disease Control and Prevention, Guangzhou, China; 4 School of Public Health, Sun Yat-Sen University, Guangzhou, China; 5 School of Public Health, Guangdong Pharmaceutica University, Guangzhou, China; 6 School of Public Health, Jinan University, Guangzhou, China; 7 Shenzhen Nanshan Center for Disease Control and Prevention, Shenzhen, China; 8 Longhua District Center for Disease Control and Prevention, Shenzhen, China

**Keywords:** Coxsackievirus A10, Hand, Foot and mouth disease, Enterovirus, Phylogenetic, Epidemiology

## Abstract

Hand, Foot and Mouth Disease (HFMD) is a highly contagious viral illness primarily affecting children globally. A significant epidemiological transition has been noted in mainland China, characterized by a substantial increase in HFMD cases caused by non-Enterovirus A71 (EV-A71) and non-Coxsackievirus A16 (CVA16) enteroviruses (EVs). Our study conducts a retrospective examination of 36,461 EV-positive specimens collected from Guangdong, China, from 2013 to 2021. Epidemiological trends suggest that, following 2013, Coxsackievirus A6 (CVA6) and Coxsackievirus A10 (CVA10) have emerged as the primary etiological agents for HFMD. In stark contrast, the incidence of EV-A71 has sharply declined, nearing extinction after 2018. Notably, cases of CVA10 infection were considerably younger, with a median age of 1.8 years, compared to 2.3 years for those with EV-A71 infections, possibly indicating accumulated EV-A71-specific herd immunity among young children. Through extensive genomic sequencing and analysis, we identified the N136D mutation in the 2 A protein, contributing to a predominant subcluster within genogroup C of CVA10 circulating in Guangdong since 2017. Additionally, a high frequency of recombination events was observed in genogroup F of CVA10, suggesting that the prevalence of this lineage might be underrecognized. The dynamic landscape of EV genotypes, along with their potential to cause outbreaks, underscores the need to broaden surveillance efforts to include a more diverse spectrum of EV genotypes. Moreover, given the shifting dominance of EV genotypes, it may be prudent to re-evaluate and optimize existing vaccination strategies, which are currently focused primarily target EV-A71.

**Supplementary Information:**

The online version contains supplementary material available at 10.1186/s12985-024-02389-9.

## Introduction

Hand, foot and mouth disease (HFMD) is a highly infectious disease predominantly affecting children worldwide, with a significant disease burden observed in the Asia-Pacific region [1]. Since 2008, mainland China has experienced a notable increase in HFMD incidence, with the incidence and mortality rate ranged from 37.01 to 205.06 and 0.0006 to 0.0142 cases per 100,000 individuals annually. Historically, Enterovirus A71 (EV-A71) and Coxsackievirus A16 (CVA16) have been identified as the main etiological agents in mainland China [2]. However, recent molecular surveillance has revealed a rising incidence of non-EV-A71/CVA16 enteroviruses (EVs).

Coxsackievirus A10 (CVA10), once infrequently identified, has increasingly been reported as a causative agent of HFMD on a global scale in recent years [3–5]. CVA10-associated HFMD have been documented in various regions, including France in 2010 [6], India in 2009–2010 [7], Thailand in 2008–2013 and 2016 [3, 8], and Singapore in 2008 and 2014 [4, 9]. Typically, CVA10 infections manifest as benign illnesses. However, severe complications such as aseptic encephalitis, cardiopulmonary failure, and death can occur [10]. Recent surveillances has highlighted a surge in severe CVA10-related cases, particularly in mainland China [11, 12], For instance, CVA10 was associated with 18.25% cases of severe HFMD cases in Jinan city, Northern China [13], and 39% in Xiamen city, Southeastern China [14]. Notably, in Guangdong Province, the most populous region in China, the number of severe HFMD cases attributed to CVA10 exceeded those linked to both CVA6 and CVA16 in 2018.

CVA10 belongs to species Enterovirus A within the family Picornaviridae. The virus genome comprises a single open reading frame (ORF), which can be divided into three precursor proteins: P1, P2 and P3 [15]. The P1 region encodes four capsid proteins (VP4, VP2, VP3 and VP1) [16]. The VP1 sequence is widely used for enterovirus-specific serotyping and genotyping [17]. Based on homology analysis of VP1 sequence, CVA10 has been classified into seven genogroups (A–G) [11, 18]. In mainland China, genotype B and genotype C are the main prevalent genotypes of CVA10. Genotype B was predominantly prevalent during 2004–2009, while genotype C has become the dominant genotype after 2009 [19].

The escalating epidemic activity of CVA10 underscores the imperative need for sustained vigilance in monitoring its epidemiology and genetic characteristics. Presently, the public database contains a limited number of CVA10 genomic sequences, and the correlation between genetic variability and shifts in epidemiological trends remains largely unexplored. In this study, we conducted a retrospective analysis of the epidemiology and genetic diversity of CVA10 in Guangdong, China, spanning from 2013 to 2021. Our findings yield valuable insights into the patterns of virus circulation and evolution, thereby enriching the global understanding of CVA10 molecular epidemic.

## Materials and methods

### HFMD Surveillance, Sample Collection and Molecular Testing

**Fig. 1:**
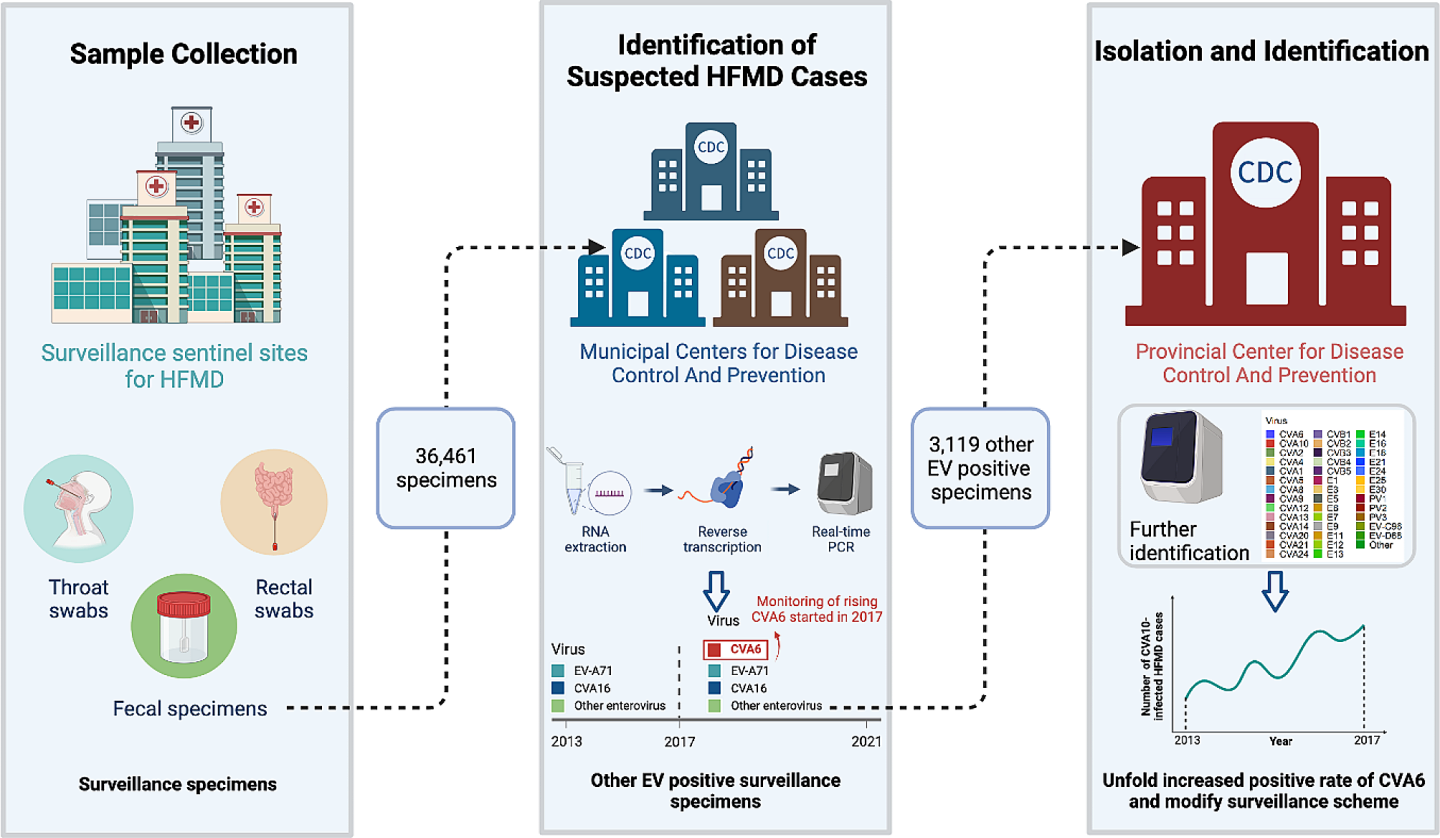
Workflow of the Guangdong Provincial HFMD surveillance Network. Medical institutions were responsible for reporting clinical cases and collecting specimens. Twenty-one Municipal Centers for Disease Control (CDCs) collected anal swabs or stool samples weekly from suspected HFMD cases and carried out primary molecular testing. From 2013 to 2016, RT-PCR testing was undertaken to detect enteroviruses, EVA71 and CVA16, and CVA6 targeted testing had been included since 2017. The specimens that tested positive for enteroviruses but negative for EVA71 and CVA16 from the years 2013–2016, as well as those negative for CVA6 from 2017–2021 were sent to the provincial CDC for further virus isolation and genetic sequencing

Hand, Foot and Mouth Disease (HFMD) is classified as a Category C notifiable disease in mainland China, and a national HFMD surveillance network was established in 2008. As previously described [20], a three-tier laboratory surveillance network was set up in Guangdong Province (Fig. 1). Medical institutions at all levels in Guangdong were responsible for reporting clinical cases and collecting specimens. HFMD cases were identified according to the Ministry of Health diagnostic criteria. Twenty-one municipal Centers for Disease Control (CDCs) collected at least eight specimens weekly from suspected HFMD cases during the epidemic season, extending from April to November. During the non-epidemic periods, from January to March and in December, the collection criteria were lowered to a minimum of three specimens per week. If the number of HFMD cases did not meet the requirement, all available cases were to be included for sample collection.

The municipal CDCs conducted primary molecular testing on a weekly basis. From 2013 to 2016, RT-PCR testing was performed to detect EV, EV-A71 and CVA16. In response to the surge of CVA6 cases in 2013, the genotyping protocol was revised in 2017 to incorporate testing for CVA6 in all HFMD samples. Specimens that tested positive for EV but negative for EV-A71 and CVA16 from 2013 to 2016, as well as those negative for CVA6 from 2017 to 2021 were sent to the provincial CDC. According to the surveillance protocol of HFMD in Guangdong Province, the provincial CDC was responsible for further virus isolation and genetic sequencing. Once the viral strains were successfully isolated, random sampling was performed based on the epidemiology prevalence and collation location. The number of HFMD cases collected for molecular testing in this study were detailed in Supplementary Table 1.

### Virus isolation and genetic sequencing

Viral strains were isolated by using rhabdomyosarcoma (RD) cells. The isolates were harvested for viral RNA extraction and sequencing. Viral RNA was extracted from the virus culture or the clinical samples with a QIAamp Mini Viral RNA Extraction Kit (Qiagen, USA) according to the manufacturer’s instructions. One step RT-PCR was performed (Qiagen, USA). The VP1 sequence was amplified by using specific primers as listed below: CVA10-VP1-F: 5’-GGAGARGCATATATTATTGCAATG-3’, CVA10-VP1-R: 5’-ACCAGGTTRGCCCAGTCAT-3’. The amplified products were further sequenced using Sanger method. For the whole genome sequencing of CVA10, a primer walking strategy was employed. Various primer pairs, detailed in Supplemental Table 2, were utilized to amplify the viral genome.

For samples positive for enteroviruses but negative for EVA71 and CVA16 (2013–2016) or negative for EVA71, CVA16, and CVA6 (2017–2021) with Ct values above 30, virus isolation was performed. This resulted in the isolation of 50, 44, 77, 64, 61, 305, 18, 23, and 66 CVA10 strains from 2013 to 2021, respectively. To account for the increased number of CVA10 cases in 2018, 33 isolates were selected from that year, while 10 isolates were chosen from each of the other years. Finally, whole genome sequences were successfully obtained for 1, 8, 9, 7, 31, 7, 7, and 8 CVA10 strains using a primer-walking sequencing strategy in 2010 and 2015–2021.

### Phylogenetic analysis

For phylogenetic reconstruction, the complete or near complete CVA10 genome sequences (7,233-7,331 nt) generated in this study (Supplementary Table 3) were combined with all publicly available CVA10 genome sequences (> 7,200 nt) in GenBank with known sampling dates (Supplementary Table 4). A total of 402 CVA10 genome sequences were used in the analysis, with 78 from this study. Multiple alignment was performed by using Mafft [21] by using AY421767 (the protype virus genome) as the reference. Using SeqKit [22], the VP1, P1, P2 and P3 region were stratified from the aligned genome sequences based on the annotation of AY421767 (VP1: 2437–3330, P1: 745–3330, P2: 3331–5064, P3: 5065–7323). Maximum-likelihood (ML) trees were estimated using IQ-TREE [23]. Molecular-clock phylogeny was inferred by assessing the accumulation of genetic changes over time via root-to-tip regression in TreeTime. Ancestral reconstruction and molecular-clock phylogeny were also estimated using TreeTime’s joint maximum likelihood analysis [24]. The annotated data and metadata were compiled and exported in JSON format for visualization in the Auspice interactive phylodynamic tool [25] (https://auspice.us/). The processing pipeline has been encapsulated within the “IPH-Nano” (registration No. 2021SR0008387) software suite for streamlined application. Bootscanning recombination analysis were performed using the Simplot program (version 3.5.1) [26]. The non-CVA10 EVs genome sequences downloaded from Genbank database were detailed in Supplemental Table 5.

### Statistical analysis

All statistical analysis was done using SPSS 19.0 (Chicago, USA). Groups comparisons were performed using Mann–Whitney test or chi-squared test. A significance threshold of *P* < 0.05 was applied to determine statistical significance. Detailed summaries of the statistical analyses are provided in Supplementary Table 6.

## Results

### Epidemiology of HFMD in Guangdong, China, 2013–2021

**Fig. 2:**
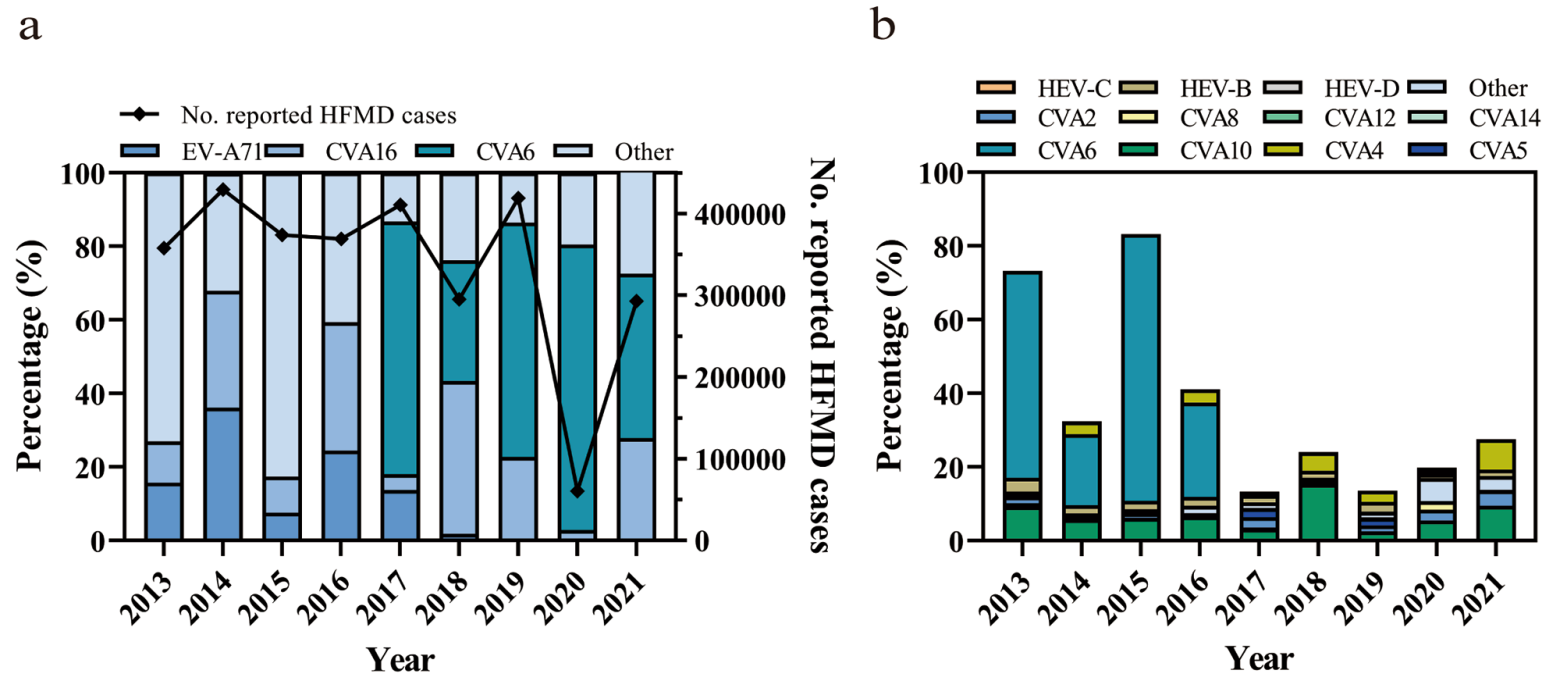
Enterovirus distribution in HFMD cases in Guangdong, China, 2013–2021. (**a**) The EVA71 and CVA16 distribution in 2013–2016, and EVA71, CVA16 and CVA6 genotypes distribution in 2017–2021; (**b**) The enterovirus genotype distributions in other enterovirus positive (non-EVA71 and non-CVA16 in 2013–2016; non-EVA71, non-CVA16 and non-CVA6 HFMD cases in 2017–2021) HFMD cases. EV, enterovirus; CV, Coxsackievirus; HFMD, Hand, foot and mouth disease

The HFMD reporting, sample collection and the molecular typing scheme was illustrated in Fig. 1. According to the provincial notifiable disease report system, a total of 3,009,233 clinically confirmed HFMD cases were reported in Guangdong, China, 2013–2021, with the highest number of cases reported in 2014 (*n* = 429,617) and the lowest in 2020 (*n* = 60,501) (Fig. 2a). RT-PCR testing for EV and specific genotypes EV-A71, CVA16 were required for collected HFMD suspicious samples in 2013–2016, which were extended to EV-A71, CVA16 and CVA6 in 2017. A total of 36,461 HFMD suspicious samples were collected for genotyping during 2013–2021, of which 26,086 (71.54%, 26,086/36,461) were detected as positive for EV. For EV genotype distribution, the ratio of EV-A71 and CVA16 were fluctuated over time. An increased CVA16 epidemic was observed every two years with a relative higher ratio (> 25%) detected in 2014, 2016, 2018 and 2021, respectively (Fig. 2a). In contrast, the low positive rate (< 1.33%) of EV-A71 was observed after 2018. Only 1 and 4 EV-A71 positive samples detected in 4,644 and 6,583 HFMD suspicious samples collected in 2020 and 2021, respectively, suggesting an extremely low circulation of this genotype in the local population.

Different from the etiology pattern of HFMD observed in 2008–2013, an increasing detection of EV genotypes other than EV-A71 and CVA16 had been noted since 2013. Our previous reports highlighted the predominance of CVA6 in HFMD cases in Guangdong by the end of 2013 [20, 27]. The sustained high incidence of CVA6 from 2013 to 2021 indicated that this genotype had become endemic in the region. Among the 3,119 samples identified as non-EV-A71/CVA16/CVA6 EVs, CVA10 constituted the largest proportion between 2013 and 2021, peaking in 2018 with a prevalence of 64.60% (500/774). Notably, CVA4 exhibited a significant upward trend, reaching the highest detection rate in 2021 at 29.82% (243/815). Additionally, CVA2, CVA5, and CVA8 were commonly detected in HFMD suspected cases, indicating a multifaceted etiological pattern of HFMD in Guangdong, China (Fig. 2b).

**Fig. 3:**
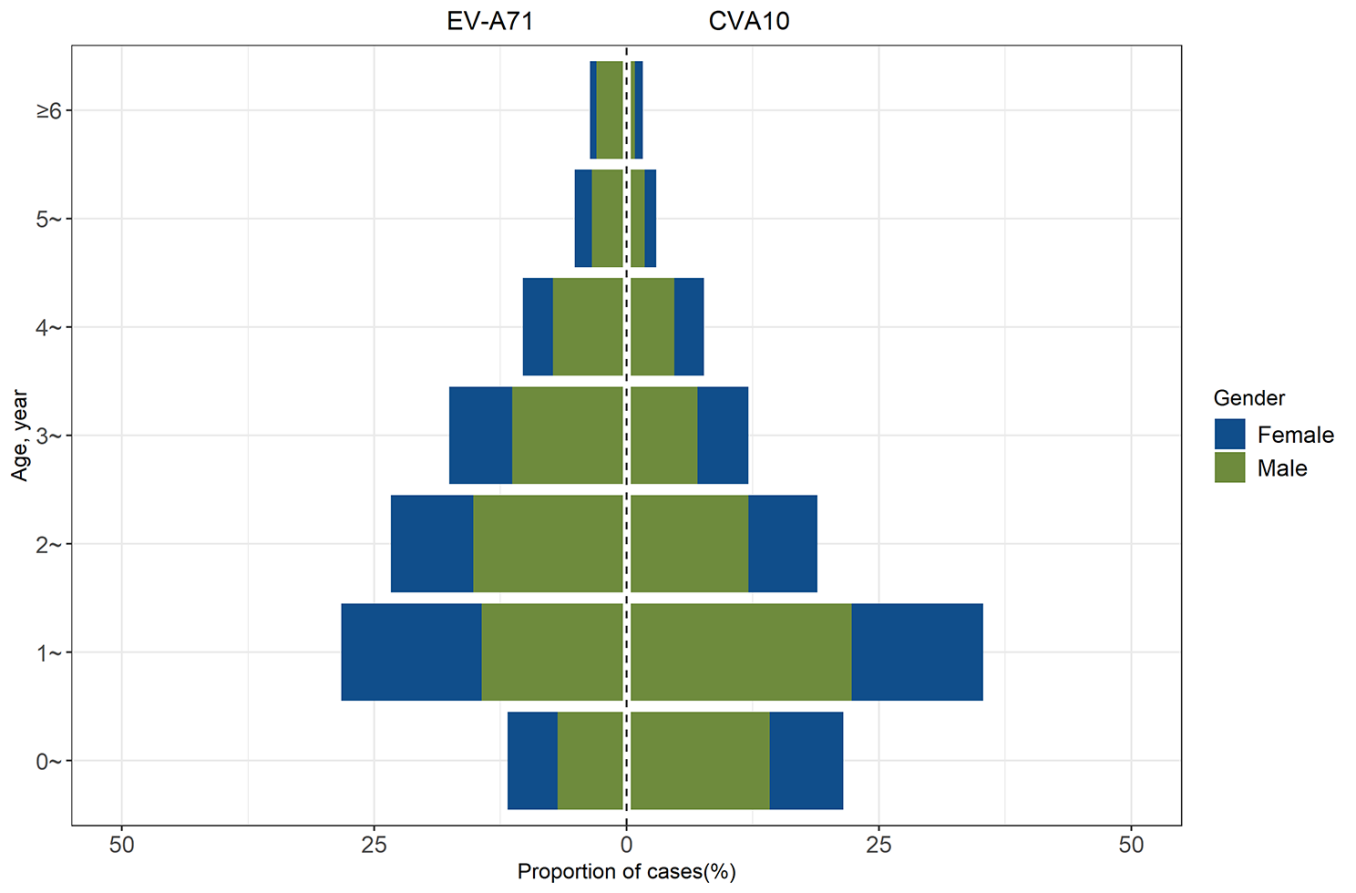
The age and gender distribution of EVA71 and CVA10 infected HFMD cases from 2017 to 2021

The prevalence of CVA10 from 2013 to 2021 had spurred an investigation into the epidemiological characteristics of CVA10 infections. We conducted a retrospective analysis of 614 cases of CVA10 infections and 467 cases of EV-A71 infections reported in the provincial notifiable disease surveillance system between 2017 and 2021. An overwhelming majority, 98.37% (604/614) of CVA10 and 96.36% (450/467) of EV-A71 infection cases, occurred in children under five years of age. Notably, the highest incidence rates for both CVA10 and EV-A71 infections were observed in the 1- to 2-year-old cohort. The median age for patients with CVA10 was 1.8 years, significantly lower than the median age of 2.3 years for EV-A71 infections (*P* < 0.05). The gender distribution showed a male-to-female ratio of approximately 1.69 for CVA10 and 1.59 for EV-A71 infections, with no significant difference between the two (*P* > 0.05), as illustrated in Fig. 3.

### Phylogenetic structure of VP1 gene and viral genome

To date, there were only a limited number of genome sequences, as well as the VP1 gene sequences, of CVA10 in public database. In order to gain the insight into the phylogenetic distribution of viruses and mutations potentially associated with the increasing epidemic, we generated a total of 78 complete or near-complete CVA10 viral genome sequences in our study, representing the samples collected from 2010 to 2021. Phylogenetic analysis was conducted by including 402 CVA10 genome sequences available from public database.

**Fig. 4:**
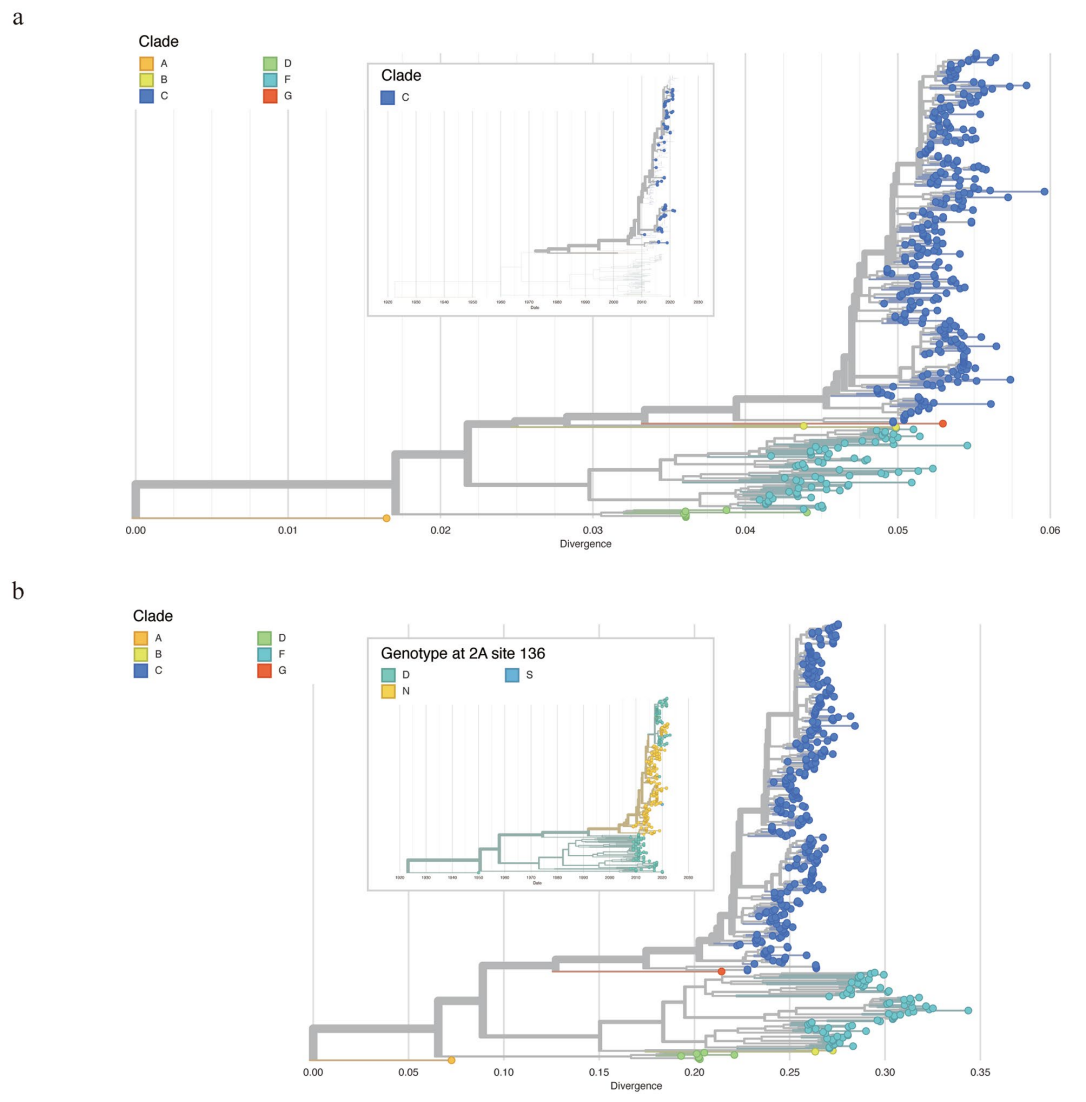
Maximum likelihood tree of CVA10 constructed based on VP1 (a), complete genome (b). The zoomed-out image in the upper panel of (**a**) illustrated the time scaled phylogenetic tree of VP1 and only Guangdong sequences generated in this study were displayed. The zoomed-out image in the upper panel of (**b**) illustrated the time scaled phylogenetic of CVA10 genome. The tree was colored according to the amino acid at 136th site of 2 A protein

Firstly, all CVA10 genome sequences were aligned to AY421767 as the reference genome. The corresponding VP1 gene were then stratified (2477-3377nt) and used for phylogenetic construction. We followed the VP1 classification scheme from the previous studies [28, 29], and all sequences could be classified into six genogroups. The VP1 gene similarities between different genogroups ranged from 74.94 to 86.47%, with genotype group A and D showing the greatest divergence (Table 1). Endemic circulation was observed in the phylogeny, with all Guangdong CVA10 genome sequences belonging to genogroup C. According to the phylogeny, the increasing prevalence of CVA10 was most likely caused by the two subcluster of genogroup C viruses representing the largest genetic cluster after 2015 (Fig. 4a, upper panel).

**Table 1:** Nucleotide identity between different genegroups of CVA10

Genogroup	Nucleotide identity (%)					
	A	B	C	D	F	G
A	100					
B	75.28–76.29	82.77–100.00				
C	75.39–77.41	80.20-85.01	83.91–100.00			
D	74.94–76.62	79.42–81.21	78.30-82.33	92.56–100.00		
F	75.17–78.75	79.75–86.47	80.09–84.90	79.64–83.22	81.85–100.00	
G	75.28	82.77–84.45	83.11–86.13	79.75–80.65	80.31–83.22	100

Secondly, we reconstructed the phylogeny by using CVA10 genome sequences and the clade was annotated according to the VP1 classification results (Fig. 4b). Ancestral sequence reconstruction with maximum likelihood method illustrated the most possible ancestral sequences for each node and the corresponding amino acid mutations occurring at each branch. Interestingly, the ancestral sequence reconstruction revealed the internal branch giving rise to the newly emerged sub-cluster of group C, encompassing the majority of CVA10 sequences gathered post-2017 (84 in 202, 41.58%), harbors a unique amino acid mutation, N136D, in the 2 A protein. This non-synonymous mutation stands in stark contrast to the 104 synonymous mutations identified across the entire genome sequences. (Fig. 4b).

Notably, the amino acid at 136th site was frequently altered during the virus evolution. The internal branch leading to the genogroup C of CVA10 including the N136D mutations and the sequences from all other genogroups were aspartic acid (Asp, D) at the 136th site of 2 A protein. The reverse mutation could be observed in the new emerged sub-cluster of group C as mentioned above. Additionally, we observed that a small group in the newly emerged cluster had N136D mutation in 2 A protein, accompanied by other amino acid mutations in the 3 A, 3 C and 3D proteins. The biological function of the mutations at 136th site of 2 A protein should be further analyzed to understand the evolution mechanism of CVA10 virus.

### Frequent recombination among CVA10 and other enterovirus a viruses

**Fig. 5:**
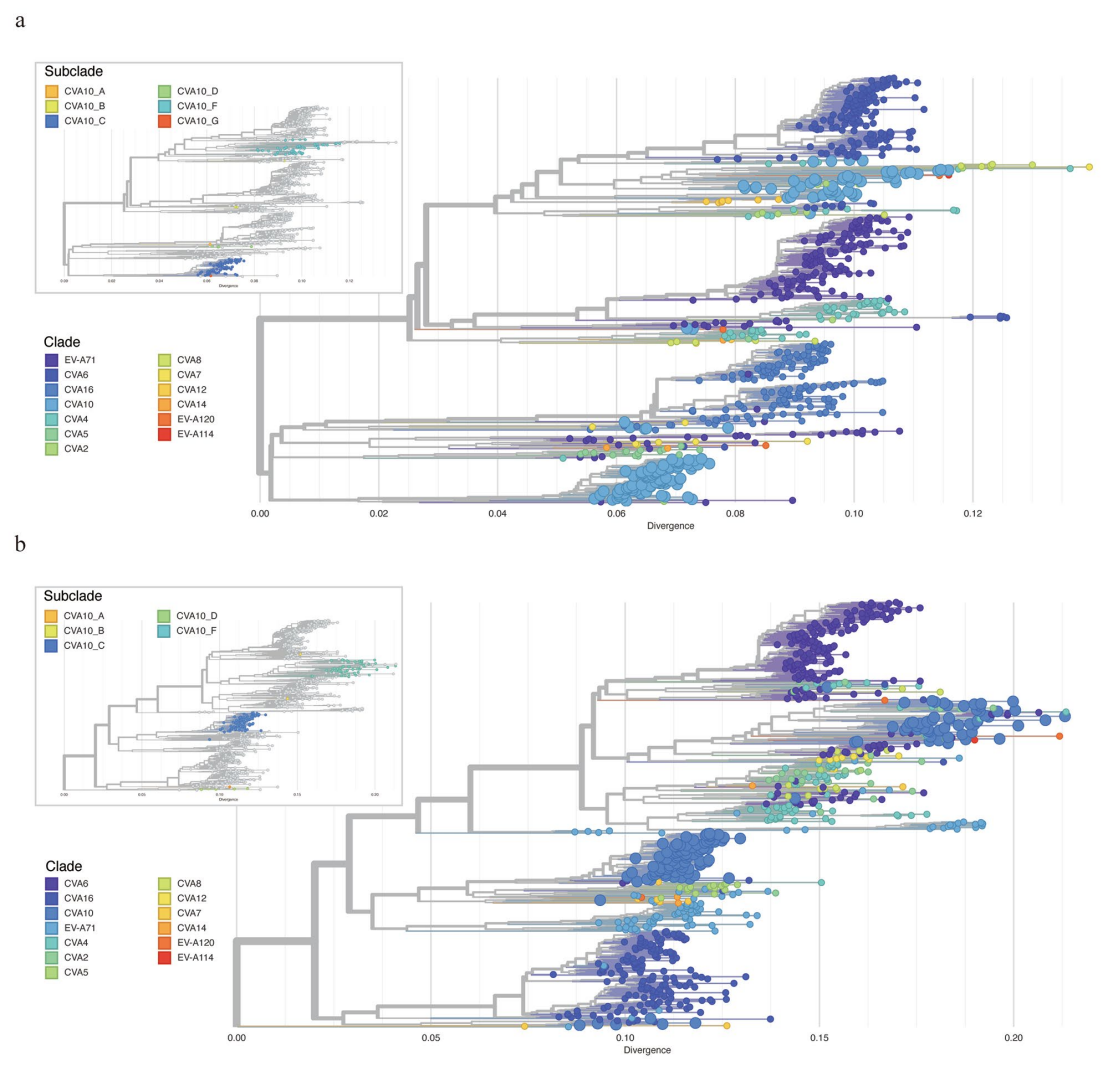
Maximum likelihood trees were reconstructed by including P2 (**a**) and P3 (**b**) region of CVA10 and other closely related enterovirus sequences. The zoomed-out panels illustrated the phylogenetic location of CVA10 sublineages

**Fig. 6:**
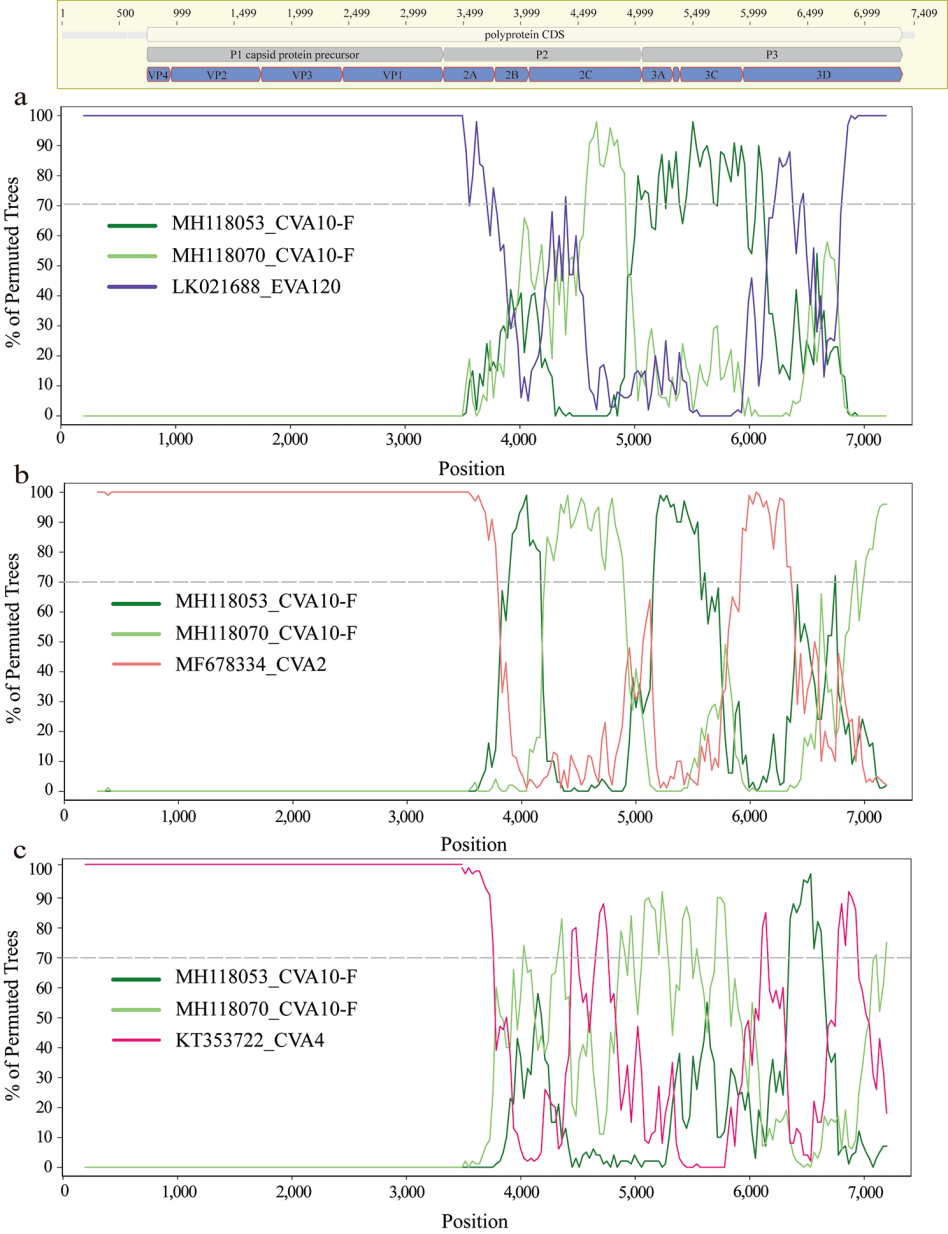
Recombination analysis of genogroup F of CVA10. Bootscanning recombination analysis between genogroup F of CVA10 and EVA120 (**a**), CVA2(**b**), CVA4(**c**). The representative strain (EVA120: MT081367, CVA2: MF422537, CVA4: OM417121) were used as query sequences respectively. The dashed line indicates > 70% bootstrap support. The genome structure of the CVA10 was annotated according to the CVA10 reference strain AY421767.

The genogroup annotations on VP1 sequences and whole genome sequences of CVA10 revealed the difference in phylogenetic structure of these two (Fig. 4a-b). In detail, the genogroup B of CVA10 sequences were closely related with genogroup G and genogroup C in phylogeny of VP1 but were clustered with genogroup F strains in phylogeny of genome sequences. This discrepancy highlighted the potential for intra-genogroup recombination or inter-EV species recombination during the evolution of the CVA10 virus. To investigate potential clues for genetic recombination, we stratified the sequences of P1, P2 and P3 regions of CVA10 genome respectively. Similarly, the genogroup G of CVA10 were more closely related with genogroup F in phylogeny of non-structure protein coding sequences (P2 and P3) which was inconsistent with the phylogeny of structure protein coding region (P1) (Supplemental Fig. 1a-c). Recombination was known to be frequent among species of EV in their non-structure protein coding regions [30, 31]. To elucidate the relationship between CVA10 and other EV species, we reconstructed the phylogeny of P2 and P3 by including all closely related sequences from other EVs. For P2 region, all genogroups of CVA10 were dispersed throughout the phylogenetic tree and clustered with other EV species rather than with other genogroup CVA10 sequences, highlighting the potential for inter-species recombination (Fig. 5a). Specifically, the P2 fragments of genogroup F fell into three different main clusters, indicating potential recombination between CVA10 genogroup F and CVA2, CVA4, CVA8, EVA114, EVA120 and other species. Genogroup C, the predominant CVA10 strains in mainland China, were all clustered together, with only one sequence (MF422532) possibly having recombination with EV-A71 and CVA2 species. Similar results were found for P3 region (Fig. 5b), where genogroup F sequences were closely related with other EV species and clustered into multiple clusters. The complex interactions between genogroup F of CVA10 and other EVs in non-structure protein coding sequences indicated the possible wide circulation of this genogroup in the population. To confirm the existence of recombination events in genogroup F of CVA10, Simplot analyses were conducted with the other EVs (Fig. 6). The results revealed multiple recombination events between genogroup F of CVA10 and EVA120, CVA2 and CVA4 in the P2 coding regions with bootstrap values over 70%.

## Discussion

HFMD is an acute contagious disease caused by a variety of human EVs, and has posed a significant burden in the Asia-Pacific region since 1997 [32]. In this retrospective study, we analyzed 36,461 EV-positive specimens collected from Guangdong, China, from 2013 to 2021. Our research highlighted a notable shift in the epidemiological landscape of HFMD in mainland China, with a marked increase in the incidence of CVA10, particularly among infants and young children aged 0 to 1 years. Genome sequencing and subsequent analysis had revealed a high frequency of recombination events within the genogroup F of CVA10 strains. Ancestral sequence reconstruction suggested a potential link between a specific mutation in the 2 A protein and the escalating epidemic of CVA10. These insights underscored the importance of continued vigilance in molecular surveillance and carried significant implications for future vaccination strategies.

Our study found the HFMD cases was highest in 2014 and lowest in 2020 similar with the epidemic trend previously observed in center of China (Nanchang) and in eastern China (Shanghai) [19, 32]. The decrease in cases in 2020 may be related to the effective implementation of non-pharmaceutical interventions during the prevention and control of COVID-19. Contrary to the rising incidence of CVA6 and CVA10 after 2013, our findings revealed that EV-A71, which previously dominated the genotype landscape in Guangdong, China, was seldom detected in 2020 and 2021. Our prior seroepidemiological research indicated that both the seroprevalence and geometric mean titer among children under three years of age increased, surpassing those in the 3–5-year age group in 2019 and 2021 [33]. The observed trend indicated that enhanced immunity within the vulnerable demographic may be contributing to a decline in the prevalence of EV-A71 infections. In our current study, the median age of patients with CVA10 infection (1.8 years old) was significantly younger than those with EV-A71 (2.3 years old) (*P* < 0.05). This age discrepancy could also reflect of increased immunity to EV-A71 among the young, vulnerable population in Guangdong, potentially due to the EV-A71 vaccination initiative that began in 2016.

Prior to this study, the scarcity of CVA10 genome sequences in public databases posed a challenge to comprehensively understand the virus’s genetic diversity and phylogenetic relationships. This study generated 78 new CVA10 genomes spanning from 2008 to 2021 and provided valuable insights into the virus’s evolution. Integrating the newly sequenced CVA10 genomes with existing data, our phylogenetic analysis unveiled intriguing patterns of inconsistency within the phylogenetic structure of different genomic regions. These findings suggested that CVA10 had undergone frequent recombination events throughout its evolutionary history, leading to a complex phylogenetic landscape.

In particular, for the P2 region of genogroup F CVA10, the phylogeny revealed that closely related sequences stemming not only from within the genogroup but also from other EV genotypes, including CVA4, CVA2, EVA114, and EVA120. This inter-genotypic relationship had led to the fragmentation of genogroup F CVA10 into several distinct phylogenetic clusters. The predominant strains of genogroup F were currently reported mainly in India [34] (Fig. 5a). This contrasts with the genogroup C of CVA10 viruses, which formed a single cluster and were primarily identified in mainland China. The complex phylogeny structure observed in P2 and P3 regions of genogroup F viruses, along with its potential for recombination with other EV genotypes, suggested that its prevalence might be largely underestimated.

In addition, through meticulous phylogenetic analysis of the CVA10 genome sequences, we had uncovered a specific mutation of 2 A protein (N136D) within the internal branch leading to the emerging subcluster of genogroup C. Notably, this mutation had become dominant in CVA10 strains identified after 2017. The 2 A protein plays a crucial role in the EV life cycle, including viral RNA replication and host cell shut-off processes [35, 36]. Therefore, the mutation at the 136th site of the 2 A protein may have significant biological implications. The fact that this mutation was maintained in genogroup C indicates that it may confer an evolutionary advantage or be linked to a functional adaptation. To fully understand the evolutionary mechanism of the CVA10 virus, further functional analyses are required. Investigating the biological consequences of the N136D mutation on the 2 A protein’s function will shed light on how this alteration may influence the virus’s replication, pathogenicity, and its interaction with host cellular machinery. Recent studies on the sequence analysis of CVA10 had mainly focused on the partial VP1 genomic region. Our study results underscored the necessity for continued molecular surveillance of EVs, with a focus on the full genomic diversity.

Given the dynamic nature of EV genotypes and their ability to cause widespread outbreaks, it is imperative that public health policies adapt to the evolving landscape of EV genotypes. Enhanced surveillance, encompassing a broader range of EV genotypes, is essential for accurately monitoring of HFMD trends. Furthermore, considering the distinct characteristics of EV-A71 and the emerging dominance of other EV genotypes, it may be prudent to re-evaluate and optimize existing vaccination strategies in mainland China, which are currently focused primarily on EV-A71.

### Electronic supplementary material

Below is the link to the electronic supplementary material.

Supplementary Material 1

Supplementary Material 2

Supplementary Material 3

Supplementary Material 4

Supplementary Material 5

Supplementary Material 6

Supplementary Material 7

Supplementary Material 8

## Data Availability

All sequences generated in this study have been submitted to the GenBank database (PP078926-PP079001, PP208912-PP208913). The json files for VP1, P1, P2, P3 and whole genome phylogenetic trees are available at zenodo with record 10470682 (https://zenodo.org/records/10470682).
