# Supplementary material for: Genomic epidemiology of CVA10 in Guangdong, China, 2013–2021
Source: Virol J. 2024 May 30;21:122. doi: 10.1186/s12985-024-02389-9 (PMC11140982; doi:10.1186/s12985-024-02389-9)
Supplement: Supplementary file 1 — Supplementary Material 1 [file 12985_2024_2389_MOESM6_ESM.docx]

**Supplementary Table 1-1.** Enterovirus distribution in HFMD cases

| Year | No. tested | EV-positive | EV isolation | EV serotype | | | |
| --- | --- | --- | --- | --- | --- | --- | --- |
|  |  |  |  | EV-A71 | CVA16 | CVA6 | Other EVs |
| 2013 | 1701 | 1404 | 1086 | 221 | 157 | - | 1026 |
| 2014 | 2033 | 1750 | 797 | 632 | 556 | - | 562 |
| 2015 | 2098 | 1851 | 2150 | 139 | 183 | - | 1529 |
| 2016 | 2113 | 1805 | 1310 | 441 | 631 | - | 733 |
| 2017 | 5576 | 4615 | 1972 | 634 | 197 | 3171 | 613 |
| 2018 | 5645 | 4176 | 1617 | 75 | 1735 | 1372 | 994 |
| 2019 | 6068 | 4457 | 732 | 16 | 997 | 2842 | 602 |
| 2020 | 4644 | 1867 | 553 | 1 | 52 | 1449 | 365 |
| 2021 | 6583 | 4161 | 764 | 4 | 1156 | 1859 | 1142 |
