# Supplementary material for: Genomic epidemiology of CVA10 in Guangdong, China, 2013–2021
Source: Virol J. 2024 May 30;21:122. doi: 10.1186/s12985-024-02389-9 (PMC11140982; doi:10.1186/s12985-024-02389-9)
Supplement: Supplementary file 2 — Supplementary Material 2 [file 12985_2024_2389_MOESM7_ESM.docx]

**Supplementary Table 1-2.** Enterovirus distribution in other enterovirus-positive HFMD cases

| Year | No. tested | Enterovirus serotype | | | | | | | | | | | |
| --- | --- | --- | --- | --- | --- | --- | --- | --- | --- | --- | --- | --- | --- |
|  |  | CVA2 | CVA4 | CVA5 | CVA6 | CVA8 | CVA10 | CVA12 | CVA14 | HEV-B | HEV-C | HEV-D | Other EVs |
| 2013 | 550 | 14 | - | 1 | 423 | 1 | 70 | 3 | 3 | 28 | 1 | - | 6 |
| 2014 | 338 | 1 | 36 | 1 | 200 | 7 | 61 | - | 2 | 23 | - | 1 | 6 |
| 2015 | 1449 | 22 | 1 | 7 | 1259 | 10 | 108 | 1 | - | 39 | 1 | - | 1 |
| 2016 | 472 | 4 | 42 | 5 | 295 | 1 | 76 | 1 | - | 28 | - | - | 20 |
| 2017 | 302 | 63 | 21 | 55 |  | 3 | 72 | 9 | - | 45 | - | - | 34 |
| 2018 | 774 | 8 | 164 | 20 |  | 4 | 500 | - | - | 53 | 2 | 3 | 20 |
| 2019 | 355 | 43 | 77 | 51 |  | 6 | 64 | 2 | - | 72 | 1 | - | 39 |
| 2020 | 241 | 36 | 11 | - |  | 29 | 67 | - | - | 14 | - | 10 | 74 |
| 2021 | 815 | 122 | 243 | 2 |  | 4 | 282 | - | - | 54 | - | - | 108 |

Other enterovirus-positive HFMD cases mean non-EVA71 and non-CVA16 in 2013-2016 and non-EVA71, non-CVA16 and non-CVA6 HFMD cases in 2017-2021.
