# Supplementary material for: Genomic epidemiology of CVA10 in Guangdong, China, 2013–2021
Source: Virol J. 2024 May 30;21:122. doi: 10.1186/s12985-024-02389-9 (PMC11140982; doi:10.1186/s12985-024-02389-9)
Supplement: Supplementary file 3 — Supplementary Material 3 [file 12985_2024_2389_MOESM3_ESM.docx]

**Supplementary Table 2** Primers used to amplify the viral genome

| Primer | Sequences | Nucleotide Location | |
| --- | --- | --- | --- |
|  |  | Minimum | Maximum |
| COX-HF | TTTAAAACAGCCTGTGGGTTG | 2415 | 2435 |
| COX-HR | GCCGGATAACGAACGTTTTC | 223 | 242 |
| Cox-1-F | TTCTGCGGAATCTTTGTGCG | 59 | 78 |
| Cox-1-R | CACCGGATGGCCAATCCAAT | 627 | 646 |
| Cox-2-F | ACTTTGGGTGTCCGTGTTTC | 550 | 569 |
| Cox-2-R | TTGCCACATTTTGGAGTC | 1150 | 1167 |
| Cox-3-F | CTTATGGAGAGTGGCCTGA | 1052 | 1070 |
| Cox-3-R | TCTGCGAGGACACTGGTATT | 1920 | 1939 |
| Cox-4-F | ATCCCAGCTGAGCTCAGGCC | 1064 | 1083 |
| Cox-4-R | CCTTGCAACACAGCCTGTTG | 2419 | 2438 |
| Cox-5-F | TGCCGGTATTACACACAATG | 2023 | 2042 |
| Cox-5-R | GTAACCATCATAAAACCACT | 3038 | 3057 |
| Cox-5-1-F | CAACAGGCTGTGTTGCAAGG | 2419 | 2438 |
| Cox-5-1-R | TCACATGCTTAAGCTTCATG | 3189 | 3208 |
| Cox-6-F | GGCAAACAGCAACTAACCCA | 2945 | 2964 |
| Cox-6-R | GCTCCCTCGGAACCAATCAG | 3874 | 3893 |
| Cox-7-F | CAGTGAGTATTACCCTGCTA | 3588 | 3607 |
| Cox-7-R | ATACGGTGTTTGCTCTTGAA | 4417 | 4436 |
| Cox-8-F | AGAAGCAGAGTGCATCTTGG | 4070 | 4089 |
| Cox-8-R | GTCTGGTGGAAGGGAGTA | 4540 | 4557 |
| Cox-9-F | AGTTCAAGAGCAAACACCGT | 4415 | 4434 |
| Cox-9-R | TTGGGCGCTCCAGAATATGC | 5326 | 5345 |
| Cox-10-F | TTAGTGACCTGCTTGCTAG | 5126 | 5144 |
| Cox-10-R | AGCACGCTTCCTCCATGCTC | 6222 | 6241 |
| Cox-11-F | AGGACCATGATGTACAATTT | 5788 | 5807 |
| Cox-11-R | ATTCCTTCTATGAGTGACAC | 6724 | 6743 |
| Cox-11-1-F | GTACCTCAGAATGACTTTTG | 6492 | 6511 |
| Cox-11-1-R | ATGGGAATTGATGGTCTGGT | 7077 | 7096 |
| Cox-12-F | GTCTGGTTCAGGGCTTTGGA | 6667 | 6686 |
| Cox-12-R | GACCAGATTTCTGGTGGGGT | 7346 | 7365 |
| COX-TF | ACCAGACCATCAATTCCCAT | 7077 | 7096 |
| COX-TR2 | GCTATTCCTGTTATAACAAATTTACCC | 7383 | 7409 |
| Cox-7-F2 | TCAGCTTCTCTAAACCTAGCC | 3551 | 3571 |
| Cox-8-R2 | TGACATGTCTTTGCCATCTGG | 4621 | 4641 |
