# Supplementary material for: Genomic epidemiology of CVA10 in Guangdong, China, 2013–2021
Source: Virol J. 2024 May 30;21:122. doi: 10.1186/s12985-024-02389-9 (PMC11140982; doi:10.1186/s12985-024-02389-9)
Supplement: Supplementary file 4 — Supplementary Material 4 [file 12985_2024_2389_MOESM1_ESM.docx]

**Supplemental Table 3** Complete genome of CVA10 sequences generated in this study

| Accession no. | Collection date | Country | Location | Genogroup |
| --- | --- | --- | --- | --- |
| R770_GD/CHN_2010 | 2010 | China | Dongguan, Guangdong | C |
| 2079_GD/CHN_2015 | 2015 | China | Zhaoqing, Guangdong | C |
| 1660_GD/CHN_2015 | 2015 | China | Shenzhen, Guangdong | C |
| 1438_GD/CHN_2015 | 2015 | China | Meizhou, Guangdong | C |
| 1208_GD/CHN_2015 | 2015 | China | Shenzhen, Guangdong | C |
| 1091_GD/CHN_2015 | 2015 | China | Jieyang, Guangdong | C |
| 0800_GD/CHN_2015 | 2015 | China | Dongguan, Guangdong | C |
| 0394_GD/CHN_2015 | 2015 | China | Foshan, Guangdong | C |
| 0276_GD/CHN_2015 | 2015 | China | Shenzhen, Guangdong | C |
| R548_GD/CHN_2016 | 2016 | China | Jieyang, Guangdong | C |
| R547_GD/CHN_2016 | 2016 | China | Jieyang, Guangdong | C |
| 541_GD/CHN_2016 | 2016 | China | Jieyang, Guangdong | C |
| 540_GD/CHN_2016 | 2016 | China | Jieyang, Guangdong | C |
| 490_GD/CHN_2016 | 2016 | China | Jieyang, Guangdong | C |
| 485_GD/CHN_2016 | 2016 | China | Jieyang, Guangdong | C |
| 454_GD/CHN_2016 | 2016 | China | Guangzhou, Guangdong | C |
| 450_GD/CHN_2016 | 2016 | China | Guangzhou, Guangdong | C |
| 1238_GD/CHN_2017 | 2017 | China | Zhuhai, Guangdong | C |
| 1168_GD/CHN_2017 | 2017 | China | Zhuhai, Guangdong | C |
| 1018_GD/CHN_2017 | 2017 | China | Shenzhen, Guangdong | C |
| 1014_GD/CHN_2017 | 2017 | China | Shenzhen, Guangdong | C |
| 1013_GD/CHN_2017 | 2017 | China | Shenzhen, Guangdong | C |
| 1340_GD/CHN_2017 | 2017 | China | Dongguan, Guangdong | C |
| 1123_GD/CHN_2017 | 2017 | China | Guangzhou, Guangdong | C |
| 997_GD/CHN_2018 | 2018 | China | Guangzhou, Guangdong | C |
| 965_GD/CHN_2018 | 2018 | China | Zhaoqing, Guangdong | C |
| 902_GD/CHN_2018 | 2018 | China | Foshan, Guangdong | C |
| 893_GD/CHN_2018 | 2018 | China | Foshan, Guangdong | C |
| 814_GD/CHN_2018 | 2018 | China | Maoming, Guangdong | C |
| 732_GD/CHN_2018 | 2018 | China | Huizhou, Guangdong | C |
| 726_GD/CHN_2018 | 2018 | China | Huizhou, Guangdong | C |
| 687_GD/CHN_2018 | 2018 | China | Shanwei, Guangdong | C |
| 611_GD/CHN_2018 | 2018 | China | Zhongshan, Guangdong | C |
| 594_GD/CHN_2018 | 2018 | China | Dongguan, Guangdong | C |
| 510_GD/CHN_2018 | 2018 | China | Jiangmen,Guangdong | C |
| 487_GD/CHN_2018 | 2018 | China | Zhuhai, Guangdong | C |
| 485_GD/CHN_2018 | 2018 | China | Zhuhai, Guangdong | C |
| 480_GD/CHN_2018 | 2018 | China | Dongguan, Guangdong | C |
| R673_GD/CHN_2018 | 2018 | China | Shantou, Guangdong | C |
| 963_GD/CHN_2018 | 2018 | China | Zhaoqing, Guangdong | C |
| 910_GD/CHN_2018 | 2018 | China | Zhanjiang, Guangdong | C |
| 907_GD/CHN_2018 | 2018 | China | Foshan, Guangdong | C |
| 835_GD/CHN_2018 | 2018 | China | Yangjiang, Guangdong | C |
| 790_GD/CHN_2018 | 2018 | China | Zhaoqing, Guangdong | C |
| 772_GD/CHN_2018 | 2018 | China | Zhaoqing, Guangdong | C |
| 763_GD/CHN_2018 | 2018 | China | Guangzhou, Guangdong | C |
| 617_GD/CHN_2018 | 2018 | China | Jiangmen, Guangdong | C |
| 593_GD/CHN_2018 | 2018 | China | Dongguan, Guangdong | C |
| 563_GD/CHN_2018 | 2018 | China | Zhuhai, Guangdong | C |
| 511_GD/CHN_2018 | 2018 | China | Jiangmen, Guangdong | C |
| 451_GD/CHN_2018 | 2018 | China | Zhanjiang, Guangdong | C |
| 449_GD/CHN_2018 | 2018 | China | Zhanjiang, Guangdong | C |
| 437_GD/CHN_2018 | 2018 | China | Zhanjiang, Guangdong | C |
| 433_GD/CHN_2018 | 2018 | China | Zhongshan, Guangdong | C |
| 0175_GD/CHN_2019 | 2019 | China | Jiangmen, Guangdong | C |
| 1430_GD/CHN_2019 | 2019 | China | Zhuhai, Guangdong | C |
| 1417_GD/CHN_2019 | 2019 | China | Shantou, Guangdong | C |
| 1402_GD/CHN_2019 | 2019 | China | Heyuan, Guangdong | C |
| 1155_GD/CHN_2019 | 2019 | China | Meizhou, Guangdong | C |
| 0388_GD/CHN_2019 | 2019 | China | Huizhou, Guangdong | C |
| 0387_GD/CHN_2019 | 2019 | China | Huizhou, Guangdong | C |
| 741_GD/CHN_2020 | 2020 | China | Jiangmen, Guangdong | C |
| 434_GD/CHN_2020 | 2020 | China | Jieyang, Guangdong | C |
| 337_GD/CHN_2020 | 2020 | China | Yangjiang, Guangdong | C |
| 293_GD/CHN_2020 | 2020 | China | Dongguan, Guangdong | C |
| 207_GD/CHN_2020 | 2020 | China | Yangjiang, Guangdong | C |
| 773_GD/CHN_2020 | 2020 | China | Zhongshan, Guangdong | C |
| 494_GD/CHN_2020 | 2020 | China | Zhongshan, Guangdong | C |
| 92_GD/CHN_2021 | 2021 | China | Dongguan, Guangdong | C |
| 72_GD/CHN_2021 | 2021 | China | Zhaoqing, Guangdong | C |
| 144_GD/CHN_2021 | 2021 | China | Huizhou, Guangdong | C |
| 81_GD/CHN_2021 | 2021 | China | Jiangmen, Guangdong | C |
| 148_GD/CHN_2021 | 2021 | China | Jieyang, Guangdong | C |
| 147_GD/CHN_2021 | 2021 | China | Jieyang, Guangdong | C |
| 123_GD/CHN_2021 | 2021 | China | Shaoguan, Guangdong | C |
| 122_GD/CHN_2021 | 2021 | China | Shaoguan, Guangdong | C |
| 520_GD/CHN_2016 | 2016 | China | Zhongshan, Guangdong | C |
| 560_GD/CHN_2018 | 2018 | China | Zhuhai, Guangdong | C |
