# Supplementary material for: Genomic epidemiology of CVA10 in Guangdong, China, 2013–2021
Source: Virol J. 2024 May 30;21:122. doi: 10.1186/s12985-024-02389-9 (PMC11140982; doi:10.1186/s12985-024-02389-9)
Supplement: Supplementary file 5 — Supplementary Material 5 [file 12985_2024_2389_MOESM2_ESM.docx]

**Supplemental Table 4** Complete genome of CVA10 reference sequences downloaded from Genbank database

| Accession no. | Collection date | Country | Location | Genogroup |
| --- | --- | --- | --- | --- |
| LT617117 | 2010 | FRANCE | FRANCE | D |
| LT617118 | 2010 | FRANCE | FRANCE | D |
| MH118027 | 2010 | India | India | D |
| AY421767 | 1950 | USA | USA | A |
| MZ441185 | 2019 | China | Other | C |
| MZ441186 | 2019 | China | Other | C |
| OK570247 | 2011 | Madagascar | Madagascar | C |
| OK570248 | 2011 | Madagascar | Madagascar | F |
| OK570250 | 2011 | Madagascar | Madagascar | C |
| OK570252 | 2011 | Madagascar | Madagascar | C |
| OK570260 | 2011 | Madagascar | Madagascar | F |
| MT950044 | 2015 | China | Other | C |
| MT950045 | 2015 | China | Other | C |
| MT950046 | 2009 | China | Other | C |
| MW713443 | 2016 | China | Other | C |
| ON101704 | 2014 | China | Other | C |
| MT350221 | 2017 | China | Other | C |
| LC483986 | 2011 | Viet Nam | Viet Nam | C |
| LC483987 | 2013 | Viet Nam | Viet Nam | C |
| LC483988 | 2016 | Viet Nam | Viet Nam | C |
| MT347977 | 2014 | USA | USA | C |
| MH118023 | 2017 | India | India | F |
| MH118033 | 2017 | India | India | F |
| MH118034 | 2017 | India | India | F |
| MH118035 | 2017 | India | India | F |
| MH118036 | 2017 | India | India | F |
| MH118037 | 2017 | India | India | F |
| MH118038 | 2017 | India | India | F |
| MH118039 | 2017 | India | India | F |
| MH118040 | 2016 | India | India | F |
| MH118041 | 2017 | India | India | D |
| MH118042 | 2015 | India | India | F |
| MH118043 | 2015 | India | India | F |
| MH118044 | 2013 | India | India | F |
| MH118045 | 2013 | India | India | F |
| MH118046 | 2013 | India | India | F |
| MH118047 | 2013 | India | India | F |
| MH118048 | 2013 | India | India | F |
| MH118049 | 2013 | India | India | F |
| MH118050 | 2013 | India | India | F |
| MH118051 | 2013 | India | India | F |
| MH118052 | 2013 | India | India | F |
| MH118053 | 2012 | India | India | F |
| MH118054 | 2012 | India | India | B |
| MH118055 | 2012 | India | India | F |
| MH118056 | 2011 | India | India | F |
| MH118057 | 2011 | India | India | F |
| MH118058 | 2011 | India | India | F |
| MH118059 | 2011 | India | India | F |
| MH118060 | 2011 | India | India | F |
| MH118061 | 2011 | India | India | F |
| MH118062 | 2011 | India | India | F |
| MH118063 | 2011 | India | India | F |
| MH118064 | 2011 | India | India | F |
| MH118065 | 2011 | India | India | F |
| MH118066 | 2011 | India | India | F |
| MH118067 | 2011 | India | India | F |
| MH118068 | 2011 | India | India | F |
| MH118069 | 2011 | India | India | F |
| MH118070 | 2011 | India | India | F |
| MH118071 | 2011 | India | India | F |
| MH118072 | 2010 | India | India | F |
| MH118073 | 2010 | India | India | F |
| MH118074 | 2010 | India | India | F |
| MH118075 | 2010 | India | India | F |
| MH118076 | 2010 | India | India | F |
| MH118077 | 2010 | India | India | F |
| MH118078 | 2010 | India | India | F |
| MH118079 | 2010 | India | India | F |
| MH118080 | 2010 | India | India | F |
| MH118081 | 2010 | India | India | F |
| MH118082 | 2010 | India | India | F |
| MH118083 | 2010 | India | India | F |
| MH118084 | 2009 | India | India | F |
| MH118085 | 2009 | India | India | F |
| MH118086 | 2009 | India | India | F |
| MH118087 | 2009 | India | India | F |
| MH118088 | 2009 | India | India | F |
| MH118089 | 2009 | India | India | F |
| MH118090 | 2009 | India | India | F |
| MH144590 | 2015 | India | India | F |
| MH144591 | 2011 | India | India | F |
| MH144592 | 2013 | India | India | F |
| MH144593 | 2017 | India | India | F |
| MH144594 | 2017 | India | India | F |
| MH144595 | 2011 | India | India | F |
| MH144596 | 2013 | India | India | F |
| MH144597 | 2017 | India | India | F |
| MH144599 | 2013 | India | India | F |
| MH144603 | 2013 | India | India | F |
| LT719056 | 2011 | Madagascar |  | F |
| LT719057 | 2011 | Madagascar |  | F |
| LT719058 | 2011 | Madagascar |  | C |
| LT719059 | 2011 | Madagascar |  | C |
| LT719060 | 2011 | Madagascar |  | C |
| MF422531 | 2008 | China | Taiwan | B |
| MF422532 | 2008 | China | Taiwan | G |
| KY012321 | 2014 | China | Other | C |
| KX768156 | 2014 | China | Other | C |
| KX768157 | 2014 | China | Other | C |
| KX768158 | 2015 | China | Other | C |
| KX768159 | 2015 | China | Other | C |
| KX768160 | 2015 | China | Other | C |
| KX768161 | 2015 | China | Other | C |
| KX768162 | 2015 | China | Other | C |
| KX768163 | 2015 | China | Other | C |
| KX768164 | 2015 | China | Other | C |
| KX768166 | 2015 | China | Other | C |
| KX768167 | 2015 | China | Other | C |
| KX768168 | 2015 | China | Other | C |
| KX430803 | 2014 | Viet Nam | Viet Nam | C |
| KX430804 | 2014 | Viet Nam | Viet Nam | C |
| KX430805 | 2014 | Viet Nam | Viet Nam | C |
| KX430806 | 2014 | Viet Nam | Viet Nam | C |
| KX430807 | 2014 | Viet Nam | Viet Nam | C |
| KX430808 | 2014 | Viet Nam | Viet Nam | C |
| KX430810 | 2014 | Viet Nam | Viet Nam | C |
| KP289394 | 2013 | China | Other | C |
| KP289395 | 2013 | China | Other | C |
| KP289396 | 2013 | China | Other | C |
| KP289397 | 2013 | China | Other | C |
| KP289398 | 2013 | China | Other | C |
| KP289399 | 2013 | China | Other | C |
| KP289400 | 2013 | China | Other | C |
| KP289401 | 2013 | China | Other | C |
| KP289402 | 2013 | China | Other | C |
| KP289403 | 2013 | China | Other | C |
| KP289404 | 2013 | China | Other | C |
| KP289405 | 2013 | China | Other | C |
| KP289406 | 2013 | China | Other | C |
| KP289407 | 2013 | China | Other | C |
| KP289408 | 2013 | China | Other | C |
| KP289409 | 2013 | China | Other | C |
| KP289410 | 2013 | China | Other | C |
| OR095105 | 2017 | China | Other | C |
| OR095106 | 2017 | China | Other | C |
| OR095107 | 2017 | China | Other | C |
| OR095108 | 2020 | China | Other | C |
| OR095109 | 2019 | China | Other | C |
| OR095110 | 2018 | China | Other | C |
| OR095111 | 2018 | China | Other | C |
| OR095112 | 2018 | China | Other | C |
| OR095113 | 2017 | China | Other | C |
| OR095114 | 2017 | China | Other | C |
| OR095115 | 2018 | China | Other | C |
| OR095116 | 2018 | China | Other | C |
| OR095117 | 2018 | China | Other | C |
| OR095118 | 2018 | China | Other | C |
| OR095119 | 2017 | China | Other | C |
| OR095120 | 2017 | China | Other | C |
| OR095121 | 2017 | China | Other | C |
| OR095122 | 2017 | China | Other | C |
| OR095123 | 2017 | China | Other | C |
| OR095124 | 2018 | China | Other | C |
| OR095125 | 2021 | China | Other | C |
| OR095126 | 2017 | China | Other | C |
| OR095127 | 2017 | China | Other | C |
| OR095128 | 2021 | China | Other | C |
| OR095129 | 2022 | China | Other | C |
| OR095130 | 2020 | China | Other | C |
| OR095131 | 2021 | China | Other | C |
| OR095132 | 2021 | China | Other | C |
| OR095133 | 2021 | China | Other | C |
| OR095134 | 2021 | China | Other | C |
| OR095135 | 2021 | China | Other | C |
| OR095136 | 2021 | China | Other | C |
| OR095137 | 2021 | China | Other | C |
| OR095138 | 2021 | China | Other | C |
| OR095139 | 2021 | China | Other | C |
| OR095140 | 2021 | China | Other | C |
| OR095141 | 2019 | China | Other | C |
| OR095142 | 2019 | China | Other | C |
| OR095143 | 2019 | China | Other | C |
| OR095144 | 2019 | China | Other | C |
| OR095145 | 2019 | China | Other | C |
| OR095146 | 2021 | China | Other | C |
| OR095147 | 2020 | China | Other | C |
| OR095148 | 2020 | China | Other | C |
| OR095149 | 2020 | China | Other | C |
| OR095150 | 2020 | China | Other | C |
| OR095151 | 2021 | China | Other | C |
| OR095152 | 2021 | China | Other | C |
| OR095153 | 2022 | China | Other | C |
| OR095154 | 2022 | China | Other | C |
| OR095155 | 2018 | China | Other | C |
| OR095156 | 2018 | China | Other | C |
| OR095157 | 2019 | China | Other | C |
| MT828546 | 2018 | China | Other | C |
| MT828547 | 2018 | China | Other | C |
| MT828548 | 2018 | China | Other | C |
| MT828549 | 2018 | China | Other | C |
| MW929238 | 2017 | China | Other | C |
| MW929239 | 2018 | China | Other | C |
| MW929240 | 2019 | China | Other | C |
| MW929241 | 2017 | China | Other | C |
| MW929242 | 2019 | China | Other | C |
| MW929243 | 2019 | China | Other | C |
| MW929244 | 2019 | China | Other | C |
| MW929245 | 2019 | China | Other | C |
| MW929246 | 2019 | China | Other | C |
| MW929247 | 2019 | China | Other | C |
| MW929248 | 2019 | China | Other | C |
| MW929249 | 2019 | China | Other | C |
| MW929250 | 2019 | China | Other | C |
| MW929251 | 2018 | China | Other | C |
| MW929252 | 2019 | China | Other | C |
| MW929253 | 2019 | China | Other | C |
| MW929254 | 2019 | China | Other | C |
| MW929255 | 2019 | China | Other | C |
| MW929256 | 2018 | China | Other | C |
| MW929257 | 2018 | China | Other | C |
| MW929258 | 2019 | China | Other | C |
| MW929259 | 2019 | China | Other | C |
| MW929260 | 2017 | China | Other | C |
| MW929261 | 2019 | China | Other | C |
| MW929262 | 2019 | China | Other | C |
| MW929263 | 2019 | China | Other | C |
| MW929264 | 2019 | China | Other | C |
| MW929265 | 2019 | China | Other | C |
| MW929266 | 2018 | China | Other | C |
| MW929267 | 2018 | China | Other | C |
| MW929268 | 2018 | China | Other | C |
| MW929269 | 2018 | China | Other | C |
| MW929270 | 2019 | China | Other | C |
| MW929271 | 2019 | China | Other | C |
| MW929272 | 2019 | China | Other | C |
| MW929273 | 2019 | China | Other | C |
| MW929274 | 2017 | China | Other | C |
| MW929275 | 2018 | China | Other | C |
| MW929276 | 2019 | China | Other | C |
| MW929277 | 2019 | China | Other | C |
| MW929278 | 2018 | China | Other | C |
| MW929279 | 2018 | China | Other | C |
| MW929280 | 2018 | China | Other | C |
| MW929281 | 2016 | China | Other | C |
| MW929282 | 2016 | China | Other | C |
| MW929283 | 2017 | China | Other | C |
| MW929284 | 2017 | China | Other | C |
| MW929285 | 2018 | China | Other | C |
| MW929286 | 2018 | China | Other | C |
| MW929287 | 2018 | China | Other | C |
| MW929288 | 2019 | China | Other | C |
| MW929289 | 2017 | China | Other | C |
| MW929290 | 2018 | China | Other | C |
| MW929291 | 2019 | China | Other | C |
| MW929292 | 2019 | China | Other | C |
| MW929293 | 2019 | China | Other | C |
| MW929294 | 2019 | China | Other | C |
| MW929295 | 2020 | China | Other | D |
| MW929296 | 2020 | China | Other | D |
| MW929297 | 2020 | China | Other | D |
| MW929298 | 2019 | China | Other | C |
| MW929299 | 2019 | China | Other | C |
| MW929300 | 2019 | China | Other | C |
| MW929301 | 2019 | China | Other | C |
| MZ491033 | 2019 | China | Other | C |
| MZ491034 | 2019 | China | Other | C |
| MK867822 | 2017 | China | Other | C |
| MK867823 | 2017 | China | Other | C |
| MK867824 | 2017 | China | Other | C |
| MK867825 | 2009 | China | Other | C |
| MK867826 | 2010 | China | Other | C |
| MK867827 | 2015 | China | Other | C |
| MK867828 | 2013 | China | Other | C |
| MK867829 | 2015 | China | Other | C |
| MK967667 | 2015 | China | Other | C |
| MK967668 | 2014 | China | Other | C |
| MK967669 | 2014 | China | Other | C |
| MK967670 | 2014 | China | Other | C |
| MK967671 | 2014 | China | Other | C |
| MT263729 | 2009 | China | Other | C |
| MK965226 | 2018 | China | Other | C |
| MK965229 | 2018 | China | Other | C |
| MK965239 | 2018 | China | Other | C |
| MK965241 | 2018 | China | Other | C |
| MK791147 | 2018 | China | Other | C |
| MK301475 | 2017 | China | Other | C |
| MK301476 | 2018 | China | Other | C |
| MK301477 | 2018 | China | Other | C |
| MK301478 | 2018 | China | Other | C |
| MK301479 | 2018 | China | Other | C |
| MK814854 | 2018 | China | Other | C |
| MK814855 | 2018 | China | Other | C |
| MK645898 | 2012 | China | Other | C |
| MH111058 | 2017 | Australia | Australia | C |
| MH111059 | 2017 | Australia | Australia | C |
| MH111060 | 2016 | Australia | Australia | C |
| MH111061 | 2016 | Australia | Australia | C |
| MH111062 | 2016 | Australia | Australia | C |
| MH111063 | 2017 | Australia | Australia | C |
| MH111064 | 2017 | Australia | Australia | C |
| MH111065 | 2017 | Australia | Australia | C |
| MH111066 | 2017 | Australia | Australia | C |
| KY271944 | 2016 | USA | USA | C |
| KX595287 | 2013 | China | Other | C |
| KX595288 | 2014 | China | Other | C |
| KX595289 | 2014 | China | Other | C |
| KX595290 | 2015 | China | Other | C |
| KY272007 | 2014 | China | Other | C |
| KY272008 | 2014 | China | Other | C |
| KY272009 | 2014 | China | Other | C |
| KY272010 | 2014 | China | Other | C |
| KT588920 | 2014 | China | Other | C |
| KU578126 | 2014 | China | Other | C |
| KU578127 | 2014 | China | Other | C |
| KU578128 | 2014 | China | Other | C |
| KU578129 | 2014 | China | Other | C |
| KU578130 | 2014 | China | Other | C |
| KU578131 | 2014 | China | Other | C |
| KU578132 | 2014 | China | Other | C |
| KU578133 | 2014 | China | Other | C |
| KU578134 | 2014 | China | Other | C |
| KU578135 | 2014 | China | Other | C |
| KU578136 | 2014 | China | Other | C |
| KP009574 | 2013 | China | Other | C |
| KP009575 | 2013 | China | Other | C |
| KP009576 | 2013 | China | Other | C |
| KP009577 | 2013 | China | Other | C |
| KP009578 | 2013 | China | Other | C |
| KP009579 | 2013 | China | Other | C |
| KP009580 | 2013 | China | Other | C |
| KP009581 | 2013 | China | Other | C |
| KJ641623 | 2013 | China | Other | C |
| HQ728262 | 2009 | China | Other | C |
