# Supplementary material for: Genomic epidemiology of CVA10 in Guangdong, China, 2013–2021
Source: Virol J. 2024 May 30;21:122. doi: 10.1186/s12985-024-02389-9 (PMC11140982; doi:10.1186/s12985-024-02389-9)
Supplement: Supplementary file 6 — Supplementary Material 6 [file 12985_2024_2389_MOESM5_ESM.docx]

**Supplementary Table 5** Complete genome of non-CVA10 EVs reference sequences downloaded from Genbank database

| Accession no. | Date | Country | Clade |
| --- | --- | --- | --- |
| MH744444 | 2015 | China | CVA5 |
| MH744475 | 2015 | China | CVA5 |
| LT719045 | 2011 | Madagascar | CVA5 |
| MW354744 | 2019 | Thailand | EV-A71 |
| MT081373 | 2018 | USA | EV-A71 |
| KU641504 | 2015 | Germany | EV-A71 |
| MG367594 | 2007 | Denmark | EV-A71 |
| JF738000 | 2009 | Thailand | EV-A71 |
| AB575935 | 1991 | Netherlands | EV-A71 |
| MW473684 | 1992 | China | EV-A71 |
| LC506514 | 1990 | Japan | EV-A71 |
| ON646273 | 2022 | China | EV-A71 |
| OQ842403 | 2020 | USA | EV-A71 |
| JX025559 | 2010 | Australia | EV-A71 |
| HQ647173 | 2006 | Canada | EV-A71 |
| AB575941 | 2007 | Netherlands | EV-A71 |
| AB575948 | 2010 | Netherlands | EV-A71 |
| OQ842404 | 2012 | USA | EV-A71 |
| OQ791538 | 2010 | USA | EV-A71 |
| MH111073 | 2016 | Australia | EV-A71 |
| MN749160 | 2013 | USA | EV-A71 |
| OP562189 | 2000 | Taiwan | CVA16 |
| MG957117 | 2016 | China | CVA16 |
| LT617113 | 2014 | France | CVA16 |
| MG571837 | 2015 | Venezuela | CVA16 |
| OQ791548 | 2013 | USA | CVA16 |
| KY674980 | 2016 | USA | CVA16 |
| MF189180 | 2016 | USA | CVA16 |
| OP562173 | 2001 | Taiwan | CVA16 |
| JQ746676 | 2005 | Malaysia | CVA16 |
| LT617103 | 2010 | France | CVA16 |
| KY792576 | 2012 | India | CVA16 |
| MH780757 | 2018 | India | CVA16 |
| JQ746663 | 1997 | Malaysia | CVA16 |
| JQ746674 | 2002 | Malaysia | CVA16 |
| JQ746675 | 2002 | Malaysia | CVA16 |
| LT617095 | 2008 | Germany | CVA16 |
| OP562176 | 2003 | Taiwan | CVA16 |
| KY425539 | 2010 | China | CVA16 |
| OP562191 | 2009 | Taiwan | CVA16 |
| MZ671867 | 2014 | China | CVA16 |
| MH111072 | 2017 | Australia | CVA16 |
| MZ671839 | 2010 | China | CVA16 |
| OP562194 | 2010 | Taiwan | CVA16 |
| MF678313 | 2008 | Australia | CVA16 |
| OP562179 | 2006 | Taiwan | CVA16 |
| MW999231 | 2012 | VietNam | CVA16 |
| MW999230 | 2012 | VietNam | CVA16 |
| MW999232 | 2012 | VietNam | CVA16 |
| MW999233 | 2012 | VietNam | CVA16 |
| MW999267 | 2013 | VietNam | CVA16 |
| MW999245 | 2014 | VietNam | CVA16 |
| MW999254 | 2014 | VietNam | CVA16 |
| MW999286 | 2017 | VietNam | CVA16 |
| MW999248 | 2015 | VietNam | CVA16 |
| MH111069 | 2016 | Australia | CVA16 |
| MZ671908 | 2019 | China | CVA16 |
| MN337593 | 2013 | China | CVA16 |
| MZ671880 | 2015 | China | CVA16 |
| JN674176 | 2010 | China | CVA16 |
| MZ671845 | 2010 | China | CVA16 |
| JX986741 | 2011 | China | CVA16 |
| JX068828 | 2011 | China | CVA16 |
| MZ671844 | 2010 | China | CVA16 |
| MZ671849 | 2011 | China | CVA16 |
| KY425535 | 2013 | China | CVA16 |
| KY425531 | 2011 | China | CVA16 |
| MT212000 | 2017 | China | CVA16 |
| KU163608 | 2010 | China | CVA16 |
| KJ746492 | 2010 | China | CVA16 |
| LC506458 | 2013 | VietNam | CVA16 |
| MZ491036 | 2019 | China | CVA16 |
| MN337595 | 2013 | China | CVA16 |
| MT212017 | 2016 | China | CVA16 |
| MZ671862 | 2014 | China | CVA16 |
| MT211993 | 2016 | China | CVA16 |
| MZ671904 | 2018 | China | CVA16 |
| MT211990 | 2016 | China | CVA16 |
| MT211996 | 2016 | China | CVA16 |
| MZ671907 | 2019 | China | CVA16 |
| MW030432 | 2018 | China | CVA16 |
| MZ671855 | 2013 | China | CVA16 |
| MT212033 | 2018 | China | CVA16 |
| MW036460 | 2018 | China | CVA16 |
| MT212036 | 2018 | China | CVA16 |
| MW036462 | 2018 | China | CVA16 |
| MT211998 | 2018 | China | CVA16 |
| MW030433 | 2018 | China | CVA16 |
| MZ671901 | 2018 | China | CVA16 |
| MZ671912 | 2019 | China | CVA16 |
| MW030434 | 2018 | China | CVA16 |
| KM609477 | 2012 | China | CVA8 |
| ON730873 | 2019 | China | CVA4 |
| ON730862 | 2020 | China | CVA4 |
| MT828544 | 2019 | China | CVA4 |
| MH780729 | 2016 | HongKong | CVA4 |
| MW161067 | 2018 | China | CVA4 |
| MW161070 | 2018 | China | CVA4 |
| MW161068 | 2018 | China | CVA4 |
| OP585372 | 2004 | Malaysia | EV-A71 |
| AB575927 | 1986 | Netherlands | EV-A71 |
| HQ189392 | 1978 | Hungary | EV-A71 |
| FJ357379 | 1986 | Taiwan | EV-A71 |
| KX064286 | 2015 | China | CVA6 |
| MF285679 | 2016 | China | CVA6 |
| KP289374 | 2013 | China | CVA6 |
| MK106215 | 2016 | China | CVA6 |
| MF422546 | 2008 | Taiwan | CVA4 |
| MK391067 | 2013 | China | CVA4 |
| MH780727 | 2010 | HongKong | CVA4 |
| MH086033 | 2016 | China | CVA4 |
| MH086036 | 2015 | China | CVA4 |
| MK658832 | 2017 | China | CVA4 |
| MH086043 | 2016 | China | CVA4 |
| MH086042 | 2015 | China | CVA4 |
| HM807310 | 2005 | Taiwan | EV-A71 |
| LC626880 | 2003 | Japan | EV-A71 |
| JX678885 | 2002 | China | EV-A71 |
| KF312457 | 1998 | China | EV-A71 |
| KC436265 | 2004 | HongKong | EV-A71 |
| JQ742002 | 2001 | China | EV-A71 |
| JQ742001 | 2001 | China | EV-A71 |
| KC436266 | 2005 | HongKong | EV-A71 |
| LC626893 | 2006 | Japan | EV-A71 |
| LC506513 | 2006 | Japan | EV-A71 |
| MG756701 | 2005 | Taiwan | EV-A71 |
| DQ133459 | 2004 | Taiwan | EV-A71 |
| KC436267 | 2006 | HongKong | EV-A71 |
| FJ194964 | 2008 | China | EV-A71 |
| KJ784495 | 2011 | China | EV-A71 |
| HQ423143 | 2009 | China | EV-A71 |
| GQ994989 | 2009 | China | EV-A71 |
| GQ994992 | 2009 | China | EV-A71 |
| FJ606447 | 2008 | China | EV-A71 |
| JX244184 | 2009 | China | EV-A71 |
| GU196833 | 2009 | China | EV-A71 |
| MN254979 | 2008 | China | EV-A71 |
| HQ129932 | 2006 | China | EV-A71 |
| FJ607336 | 2008 | China | EV-A71 |
| FJ360546 | 2008 | China | EV-A71 |
| FJ828519 | 2008 | China | EV-A71 |
| JX244182 | 2009 | China | EV-A71 |
| EU753397.2 | 2007 | China | EV-A71 |
| HM002486 | 2008 | China | EV-A71 |
| MK904809 | 2018 | China | EV-A71 |
| HQ694982 | 2008 | China | EV-A71 |
| OQ355809 | 2009 | China | EV-A71 |
| KJ004560 | 2010 | China | EV-A71 |
| HM053670 | 2009 | China | EV-A71 |
| HM002488 | 2009 | China | EV-A71 |
| JN052925 | 2011 | China | EV-A71 |
| MF662689 | 2011 | China | EV-A71 |
| GQ994990 | 2009 | China | EV-A71 |
| HM053671 | 2009 | China | EV-A71 |
| JQ086365 | 2011 | China | EV-A71 |
| MG756717 | 2010 | Taiwan | EV-A71 |
| KF444809 | 2012 | China | EV-A71 |
| OQ355796 | 2011 | China | EV-A71 |
| OQ355778 | 2014 | China | EV-A71 |
| KT428647 | 2014 | China | EV-A71 |
| KU936125 | 2014 | China | EV-A71 |
| KX752783 | 2015 | China | EV-A71 |
| OQ355808 | 2009 | China | EV-A71 |
| HM002489 | 2009 | China | EV-A71 |
| KP289431 | 2013 | China | EV-A71 |
| KP289428 | 2013 | China | EV-A71 |
| MG773123 | 2014 | China | EV-A71 |
| KP289417 | 2013 | China | EV-A71 |
| KU254596 | 2014 | China | EV-A71 |
| KJ686147 | 2011 | VietNam | EV-A71 |
| KP308427 | 2012 | Cambodia | EV-A71 |
| KJ686235 | 2012 | VietNam | EV-A71 |
| KJ686135 | 2012 | VietNam | EV-A71 |
| KP308441 | 2012 | Cambodia | EV-A71 |
| KJ686156 | 2012 | VietNam | EV-A71 |
| KC436272 | 2010 | HongKong | EV-A71 |
| OQ355802 | 2010 | China | EV-A71 |
| HQ891926 | 2009 | China | EV-A71 |
| KM055005 | 2011 | Laos | EV-A71 |
| KJ686189 | 2011 | VietNam | EV-A71 |
| JQ074187 | 2010 | China | EV-A71 |
| KJ004559 | 2010 | China | EV-A71 |
| MG756722 | 2011 | Taiwan | EV-A71 |
| OQ355793 | 2012 | China | EV-A71 |
| KC570453 | 2012 | China | EV-A71 |
| KJ004554 | 2012 | China | EV-A71 |
| KJ632499 | 2011 | China | EV-A71 |
| MF662695 | 2012 | China | EV-A71 |
| KJ004557 | 2010 | China | EV-A71 |
| JX244187 | 2010 | China | EV-A71 |
| MN747117 | 2010 | China | EV-A71 |
| MF662691 | 2011 | China | EV-A71 |
| MH511207 | 2017 | China | EV-A71 |
| MG756720 | 2011 | Taiwan | EV-A71 |
| MF662696 | 2012 | China | EV-A71 |
| KF142413 | 2013 | China | EV-A71 |
| OQ355788 | 2013 | China | EV-A71 |
| OP191657 | 2017 | China | EV-A71 |
| OQ355770 | 2015 | China | EV-A71 |
| OQ355769 | 2015 | China | EV-A71 |
| OQ355784 | 2013 | China | EV-A71 |
| KU936129 | 2014 | China | EV-A71 |
| ON502227 | 2016 | China | EV-A71 |
| ON502341 | 2018 | China | EV-A71 |
| ON502197 | 2017 | China | EV-A71 |
| ON502366 | 2016 | China | EV-A71 |
| OQ355761 | 2015 | China | EV-A71 |
| OQ355765 | 2015 | China | EV-A71 |
| ON502238 | 2017 | China | EV-A71 |
| OQ355751 | 2017 | China | EV-A71 |
| ON502266 | 2016 | China | EV-A71 |
| ON502350 | 2016 | China | EV-A71 |
| ON502342 | 2018 | China | EV-A71 |
| OQ355747 | 2018 | China | EV-A71 |
| OQ355760 | 2016 | China | EV-A71 |
| JQ639383 | 2011 | China | EV-A71 |
| OQ355783 | 2013 | China | EV-A71 |
| KP289425 | 2013 | China | EV-A71 |
| KJ004558 | 2010 | China | EV-A71 |
| OQ355776 | 2014 | China | EV-A71 |
| KF142412 | 2013 | China | EV-A71 |
| MK697691 | 2013 | Australia | EV-A71 |
| MF662694 | 2012 | China | EV-A71 |
| KT008671 | 2014 | China | EV-A71 |
| KU936130 | 2014 | China | EV-A71 |
| ON502309 | 2016 | China | EV-A71 |
| JQ086366 | 2011 | China | EV-A71 |
| KJ784496 | 2012 | China | EV-A71 |
| KP308406 | 2012 | Cambodia | EV-A71 |
| KT008669 | 2014 | China | EV-A71 |
| KP289423 | 2013 | China | EV-A71 |
| MF662699 | 2013 | China | EV-A71 |
| KP289418 | 2013 | China | EV-A71 |
| ON502255 | 2016 | China | EV-A71 |
| ON502329 | 2019 | China | EV-A71 |
| ON502191 | 2018 | China | EV-A71 |
| ON502346 | 2016 | China | EV-A71 |
| OQ355764 | 2015 | China | EV-A71 |
| MG875331 | 2016 | China | EV-A71 |
| ON502212 | 2016 | China | EV-A71 |
| ON502365 | 2016 | China | EV-A71 |
| ON502300 | 2016 | China | EV-A71 |
| ON502373 | 2016 | China | EV-A71 |
| OQ355768 | 2015 | China | EV-A71 |
| ON502356 | 2016 | China | EV-A71 |
| ON502310 | 2016 | China | EV-A71 |
| MG581490 | 2017 | China | EV-A71 |
| OQ355762 | 2016 | China | EV-A71 |
| ON502239 | 2017 | China | EV-A71 |
| ON502304 | 2018 | China | EV-A71 |
| ON502215 | 2018 | China | EV-A71 |
| ON502240 | 2016 | China | EV-A71 |
| OQ355756 | 2016 | China | EV-A71 |
| ON502303 | 2016 | China | EV-A71 |
| MG756691 | 2016 | Taiwan | EV-A71 |
| ON502236 | 2018 | China | EV-A71 |
| ON502291 | 2018 | China | EV-A71 |
| OQ355753 | 2017 | China | EV-A71 |
| ON502231 | 2018 | China | EV-A71 |
| ON502369 | 2018 | China | EV-A71 |
| MT708804 | 2019 | China | EV-A71 |
| ON502324 | 2019 | China | EV-A71 |
| OQ355737 | 2019 | China | EV-A71 |
| OQ355743 | 2018 | China | EV-A71 |
| MH111022 | 2016 | Australia | CVA4 |
| ON730872 | 2019 | China | CVA4 |
| MK391075 | 2016 | China | CVA4 |
| MK391071 | 2014 | China | CVA4 |
| MT814407 | 2014 | France | CVA6 |
| MT814553 | 2014 | France | CVA6 |
| KX212527 | 2014 | Denmark | CVA6 |
| KP144349 | 2011 | UnitedKingdom | CVA6 |
| KP144353 | 2013 | UnitedKingdom | CVA6 |
| MT814548 | 2016 | France | CVA6 |
| ON755022 | 2013 | China | CVA12 |
| MK061425 | 2018 | China | CVA12 |
| MH111018 | 2017 | Australia | CVA2 |
| MF422537 | 2008 | Taiwan | CVA2 |
| OM417121 | 2012 | Thailand | CVA4 |
| MT641400 | 2018 | UnitedKingdom | CVA4 |
| MG571858 | 2015 | Venezuela | CVA2 |
| MK652141 | 2018 | Venezuela | CVA7 |
| MT648787 | 2017 | China | CVA8 |
| MT648780 | 2014 | China | CVA8 |
| MT648778 | 2015 | China | CVA8 |
| MT648782 | 2018 | China | CVA8 |
| MH111021 | 2016 | Australia | CVA4 |
| KX212517 | 2014 | Thailand | CVA6 |
| MT814541 | 2014 | France | CVA6 |
| MT814545 | 2014 | France | CVA6 |
| MT814536 | 2016 | France | CVA6 |
| MT814606 | 2011 | France | CVA6 |
| MH111046 | 2016 | Australia | CVA6 |
| MT814612 | 2018 | France | CVA6 |
| KX212524 | 2014 | Denmark | CVA6 |
| MT814595 | 2016 | France | CVA6 |
| MT814575 | 2018 | France | CVA6 |
| MT814572 | 2017 | France | CVA6 |
| MT814579 | 2010 | France | CVA6 |
| LC421555 | 2008 | Japan | CVA6 |
| LC126145 | 1999 | Japan | CVA6 |
| MT814502 | 2010 | France | CVA6 |
| LC126158 | 2011 | Japan | CVA6 |
| MF285630 | 2013 | China | CVA6 |
| KP289388 | 2013 | China | CVA6 |
| MT814453 | 2013 | France | CVA6 |
| MT814523 | 2011 | France | CVA6 |
| MT814507 | 2012 | France | CVA6 |
| MT814477 | 2014 | France | CVA6 |
| MT814516 | 2011 | France | CVA6 |
| KP144343 | 2014 | UnitedKingdom | CVA6 |
| MT814581 | 2014 | France | CVA6 |
| MF285674 | 2016 | China | CVA6 |
| MH371303 | 2017 | Italy | CVA6 |
| KX212507 | 2014 | Thailand | CVA6 |
| LC421568 | 2013 | Japan | CVA6 |
| LC421571 | 2013 | Japan | CVA6 |
| MT814487 | 2014 | France | CVA6 |
| MK510082 | 2016 | USA | CVA6 |
| LC421584 | 2017 | Japan | CVA6 |
| MF285667 | 2015 | China | CVA6 |
| KP289393 | 2013 | China | CVA6 |
| KX189178 | 2011 | China | CVA6 |
| KX189180 | 2011 | China | CVA6 |
| KX212495 | 2010 | Thailand | CVA6 |
| LC126150 | 2009 | Japan | CVA6 |
| LC421562 | 2010 | Japan | CVA6 |
| MT814517 | 2014 | France | CVA6 |
| KP144342 | 2013 | UnitedKingdom | CVA6 |
| MF678318 | 2012 | Australia | CVA6 |
| MH539784 | 2015 | India | CVA6 |
| MH539787 | 2016 | India | CVA6 |
| MF578347 | 2012 | VietNam | CVA6 |
| MF578320 | 2011 | VietNam | CVA6 |
| MF578362 | 2012 | VietNam | CVA6 |
| MF578358 | 2014 | VietNam | CVA6 |
| MF578298 | 2015 | VietNam | CVA6 |
| MF578284 | 2014 | VietNam | CVA6 |
| MF578308 | 2014 | VietNam | CVA6 |
| MH111054 | 2017 | Australia | CVA6 |
| MF578311 | 2014 | VietNam | CVA6 |
| MK106198 | 2014 | China | CVA6 |
| MH086202 | 2015 | China | CVA6 |
| KX064294 | 2015 | China | CVA6 |
| MH086199 | 2015 | China | CVA6 |
| MT814414 | 2017 | France | CVA6 |
| MN845782 | 2016 | China | CVA6 |
| MF285671 | 2016 | China | CVA6 |
| MF285658 | 2015 | China | CVA6 |
| KX212494 | 2014 | Thailand | CVA6 |
| MT814519 | 2015 | France | CVA6 |
| MH111045 | 2016 | Australia | CVA6 |
| KP289378 | 2013 | China | CVA6 |
| MF285662 | 2015 | China | CVA6 |
| KP289385 | 2013 | China | CVA6 |
| MH086162 | 2015 | China | CVA6 |
| MH086163 | 2015 | China | CVA6 |
| MH086165 | 2015 | China | CVA6 |
| MH086210 | 2015 | China | CVA6 |
| MF285680 | 2016 | China | CVA6 |
| MN845824 | 2018 | China | CVA6 |
| MF285681 | 2016 | China | CVA6 |
| MN845819 | 2018 | China | CVA6 |
| MK106213 | 2016 | China | CVA6 |
| MN845783 | 2016 | China | CVA6 |
| MN845796 | 2016 | China | CVA6 |
| MN845789 | 2016 | China | CVA6 |
| MN845804 | 2017 | China | CVA6 |
| KX595285 | 2014 | China | CVA6 |
| MH086148 | 2016 | China | CVA6 |
| MN845797 | 2016 | China | CVA6 |
| MF285676 | 2016 | China | CVA6 |
| MF285683 | 2016 | China | CVA6 |
| KX064297 | 2015 | China | CVA6 |
| OL830032 | 2018 | China | CVA6 |
| OL839941 | 2018 | China | CVA6 |
| MN845815 | 2018 | China | CVA6 |
| MN845880 | 2017 | China | CVA6 |
| OL839946 | 2018 | China | CVA6 |
| OP896722 | 2022 | Thailand | CVA6 |
| OL519581 | 2018 | China | CVA6 |
| MN845856 | 2017 | China | CVA6 |
| MN845860 | 2017 | China | CVA6 |
| MN845851 | 2017 | China | CVA6 |
| MN845890 | 2018 | China | CVA6 |
| OP896715 | 2019 | Thailand | CVA6 |
| AB575912 | 1966 | Netherlands | EV-A71 |
| LT617111 | 2014 | France | CVA16 |
| LT617105 | 2011 | France | CVA16 |
| OQ791549 | 2013 | USA | CVA16 |
| MN749141 | 2015 | USA | CVA16 |
| OP562175 | 2002 | Taiwan | CVA16 |
| JQ746662 | 1999 | Malaysia | CVA16 |
| LT617099 | 2010 | France | CVA16 |
| OP562177 | 2004 | Taiwan | CVA16 |
| LT617094 | 2008 | Hungary | CVA16 |
| OP562178 | 2005 | Taiwan | CVA16 |
| OP562197 | 2012 | Taiwan | CVA16 |
| OP562198 | 2013 | Taiwan | CVA16 |
| LC506462 | 2017 | VietNam | CVA16 |
| MH111071 | 2016 | Australia | CVA16 |
| MZ491035 | 2019 | China | CVA16 |
| MF678332 | 2006 | Australia | CVA16 |
| KM055004 | 2011 | Laos | CVA16 |
| OP562180 | 2007 | Taiwan | CVA16 |
| OP562196 | 2010 | Taiwan | CVA16 |
| MZ671840 | 2010 | China | CVA16 |
| MH111070 | 2017 | Australia | CVA16 |
| KU854873 | 2008 | China | CVA16 |
| KY425538 | 2010 | China | CVA16 |
| OP562182 | 2009 | Taiwan | CVA16 |
| KC755232 | 2010 | China | CVA16 |
| MH010198 | 2014 | China | CVA16 |
| KC755229 | 2010 | China | CVA16 |
| JX068831 | 2011 | China | CVA16 |
| KF193630 | 2010 | China | CVA16 |
| KP289412 | 2013 | China | CVA16 |
| JQ746668 | 2006 | Malaysia | CVA16 |
| LT617102 | 2010 | France | CVA16 |
| KY792578 | 2013 | India | CVA16 |
| KY792577 | 2012 | India | CVA16 |
| KY792583 | 2013 | India | CVA16 |
| KY014077 | 2008 | China | CVA16 |
| JX068832 | 2009 | China | CVA16 |
| KF193627 | 2009 | China | CVA16 |
| KF193620 | 2008 | China | CVA16 |
| KF193624 | 2009 | China | CVA16 |
| MZ671838 | 2010 | China | CVA16 |
| MG674827 | 2015 | China | CVA16 |
| MT211999 | 2016 | China | CVA16 |
| KC117318 | 2009 | China | CVA16 |
| JX986740 | 2011 | China | CVA16 |
| GQ279371 | 2008 | China | CVA16 |
| JX068827 | 2010 | China | CVA16 |
| JQ034149 | 2010 | China | CVA16 |
| MZ671847 | 2011 | China | CVA16 |
| JX068830 | 2011 | China | CVA16 |
| KY425530 | 2011 | China | CVA16 |
| MZ671900 | 2018 | China | CVA16 |
| OM417117 | 2016 | Thailand | CVA16 |
| MK697682 | 2013 | Australia | CVA16 |
| MZ671857 | 2013 | China | CVA16 |
| MZ671893 | 2016 | China | CVA16 |
| MT211995 | 2018 | China | CVA16 |
| MT212005 | 2018 | China | CVA16 |
| MZ671909 | 2019 | China | CVA16 |
| MT212026 | 2016 | China | CVA16 |
| MZ671895 | 2017 | China | CVA16 |
| KP289416 | 2013 | China | CVA16 |
| KY425529 | 2015 | China | CVA16 |
| MZ671883 | 2015 | China | CVA16 |
| MZ671876 | 2014 | China | CVA16 |
| MZ671878 | 2014 | China | CVA16 |
| MT212023 | 2017 | China | CVA16 |
| MN337591 | 2013 | China | CVA16 |
| MZ671877 | 2014 | China | CVA16 |
| MT212032 | 2017 | China | CVA16 |
| MT212013 | 2016 | China | CVA16 |
| MH010199 | 2015 | China | CVA16 |
| MZ671902 | 2018 | China | CVA16 |
| MT212002 | 2018 | China | CVA16 |
| MT211997 | 2017 | China | CVA16 |
| MZ671911 | 2019 | China | CVA16 |
| MT212010 | 2018 | China | CVA16 |
| ON646272 | 2018 | China | CVA16 |
| MT212025 | 2017 | China | CVA16 |
| MT212015 | 2018 | China | CVA16 |
| AB575928 | 1986 | Netherlands | EV-A71 |
| AB575915 | 1974 | Netherlands | EV-A71 |
| AB575916 | 1976 | Netherlands | EV-A71 |
| FJ357381 | 1986 | Taiwan | EV-A71 |
| MH716380 | 2014 | VietNam | EV-A71 |
| FJ357377 | 2000 | Taiwan | EV-A71 |
| KT354875 | 2011 | Taiwan | EV-A71 |
| LC375765 | 1997 | Japan | EV-A71 |
| LC375766 | 2003 | Japan | EV-A71 |
| MN053433 | 2006 | Malaysia | EV-A71 |
| MN053434 | 2006 | Malaysia | EV-A71 |
| KR045296 | 2012 | Thailand | EV-A71 |
| JF738001 | 2009 | Thailand | EV-A71 |
| KX372312 | 2011 | Thailand | EV-A71 |
| MG756713 | 2008 | Taiwan | EV-A71 |
| MG756709 | 2008 | Taiwan | EV-A71 |
| MG756742 | 2012 | Taiwan | EV-A71 |
| JN964686 | 2009 | China | EV-A71 |
| LC321993 | 2017 | Japan | EV-A71 |
| MN966518 | 2016 | China | EV-A71 |
| KX372320 | 2012 | Thailand | EV-A71 |
| LC626874 | 2013 | Japan | EV-A71 |
| KX372331 | 2014 | Thailand | EV-A71 |
| KX372329 | 2014 | Thailand | EV-A71 |
| KX372325 | 2013 | Thailand | EV-A71 |
| OM417112 | 2016 | Thailand | EV-A71 |
| KU647000 | 2014 | China | EV-A71 |
| MH716351 | 2015 | VietNam | EV-A71 |
| LC627067 | 2015 | VietNam | EV-A71 |
| MH716356 | 2014 | VietNam | EV-A71 |
| LC627070 | 2015 | VietNam | EV-A71 |
| MH716343 | 2013 | VietNam | EV-A71 |
| MH716346 | 2013 | VietNam | EV-A71 |
| MH716368 | 2013 | VietNam | EV-A71 |
| MH716321 | 2014 | VietNam | EV-A71 |
| MH716374 | 2014 | VietNam | EV-A71 |
| MH716375 | 2014 | VietNam | EV-A71 |
| MH716304 | 2014 | VietNam | EV-A71 |
| MH716307 | 2015 | VietNam | EV-A71 |
| MH716284 | 2014 | VietNam | EV-A71 |
| MH716300 | 2015 | VietNam | EV-A71 |
| LT719046 | 2011 | Madagascar | CVA5 |
| ON191508 | 2016 | Senegal | EV-A71 |
| OP562190 | 2000 | Taiwan | CVA16 |
| MH744446 | 2015 | China | CVA5 |
| MH744474 | 2015 | China | CVA5 |
| MT081374 | 2018 | USA | EV-A71 |
| MH484069 | 2016 | Spain | EV-A71 |
| MW354743 | 2019 | Thailand | EV-A71 |
| MF662679 | 2009 | China | EV-A71 |
| JX025561 | 2008 | China | EV-A71 |
| OQ355787 | 2013 | China | EV-A71 |
| KC954663 | 2008 | China | EV-A71 |
| KM211580 | 2012 | China | EV-A71 |
| KU936121 | 2014 | China | EV-A71 |
| MN747118 | 2012 | China | EV-A71 |
| ON502297 | 2018 | China | EV-A71 |
| ON502353 | 2016 | China | EV-A71 |
| OQ355752 | 2017 | China | EV-A71 |
| ON502282 | 2018 | China | EV-A71 |
| MH086035 | 2016 | China | CVA4 |
| MH086047 | 2016 | China | CVA4 |
| MH086038 | 2016 | China | CVA4 |
| MK658831 | 2017 | China | CVA4 |
| MH086041 | 2015 | China | CVA4 |
| MH086045 | 2015 | China | CVA4 |
| HQ728260 | 2009 | China | CVA4 |
| LC421549 | 2002 | Japan | CVA6 |
| LC126144 | 1999 | Japan | CVA6 |
| LC126146 | 2003 | Japan | CVA6 |
| LC421542 | 2001 | Japan | CVA6 |
| LC421550 | 2005 | Japan | CVA6 |
| MF838736 | 2006 | Australia | CVA6 |
| MT814528 | 2010 | France | CVA6 |
| MT814534 | 2010 | France | CVA6 |
| MT814535 | 2010 | France | CVA6 |
| KX156350 | 2013 | China | CVA2 |
| JQ946050 | 2009 | Taiwan | CVA6 |
| MF422555 | 2008 | Taiwan | CVA6 |
| LC506447 | 2012 | VietNam | CVA6 |
| KM609476 | 2012 | China | CVA8 |
| MK391072 | 2015 | China | CVA4 |
| KT779411 | 2013 | China | CVA6 |
| MF373606 | 2012 | China | CVA6 |
| MH086143 | 2015 | China | CVA6 |
| MK967651 | 2018 | China | CVA6 |
| OL357660 | 2020 | China | CVA2 |
| KX372309 | 2008 | Thailand | EV-A71 |
| KM609481 | 2013 | China | CVA8 |
| MT648779 | 2013 | China | CVA8 |
| KM279379 | 2013 | China | CVA6 |
| KP289366 | 2013 | China | CVA6 |
| MK967658 | 2016 | China | CVA2 |
| MW846233 | 2017 | China | CVA2 |
| ON730867 | 2019 | China | CVA4 |
| OL519580 | 2018 | China | CVA2 |
| MN337601 | 2013 | China | CVA6 |
| KP289357 | 2013 | China | CVA2 |
| KX595281 | 2012 | China | CVA2 |
| MN964077 | 2016 | China | CVA4 |
| KP289360 | 2013 | China | CVA2 |
| KX595283 | 2013 | China | CVA2 |
| MW161069 | 2018 | China | CVA4 |
| MW267852 | 2018 | China | CVA4 |
| MW161072 | 2018 | China | CVA4 |
| KP289361 | 2013 | China | CVA2 |
| MF281257 | 2015 | China | CVA2 |
| KX595284 | 2015 | China | CVA2 |
| OL357657 | 2020 | China | CVA2 |
| OL357658 | 2020 | China | CVA2 |
| MZ491028 | 2019 | China | CVA2 |
| MZ491030 | 2019 | China | CVA5 |
| ON755036 | 2019 | China | CVA12 |
| MT350223 | 2013 | China | CVA2 |
| ON730874 | 2019 | China | CVA4 |
| ON730853 | 2020 | China | CVA4 |
| ON730857 | 2019 | China | CVA4 |
| ON730864 | 2020 | China | CVA4 |
| OL357659 | 2019 | China | CVA2 |
| ON730865 | 2019 | China | CVA4 |
| EU812514 | 2008 | China | CVA16 |
| JQ950555 | 2010 | Australia | EV-A71 |
| ON755032 | 2013 | China | CVA12 |
| ON755029 | 2015 | China | CVA12 |
| MK061424 | 2018 | China | CVA12 |
| MT814556 | 2014 | France | CVA6 |
| MT814549 | 2016 | France | CVA6 |
| MT814557 | 2010 | France | CVA6 |
| KP765687 | 2014 | China | CVA8 |
| MT648788 | 2017 | China | CVA8 |
| MT814591 | 2014 | France | CVA6 |
| MT814573 | 2018 | France | CVA6 |
| MT814602 | 2016 | France | CVA6 |
| MT814597 | 2016 | France | CVA6 |
| MT814605 | 2018 | France | CVA6 |
| MT814570 | 2018 | France | CVA6 |
| MT814583 | 2018 | France | CVA6 |
| MT814580 | 2010 | France | CVA6 |
| MF422539 | 2008 | Taiwan | CVA2 |
| MT814608 | 2011 | France | CVA6 |
| HM622391 | 2008 | Taiwan | EV-A71 |
| KX372340 | 2012 | Thailand | CVA6 |
| MT814538 | 2014 | France | CVA6 |
| MT814543 | 2014 | France | CVA6 |
| MH111039 | 2016 | Australia | CVA6 |
| MT814611 | 2017 | France | CVA6 |
| MH111028 | 2017 | Australia | CVA4 |
| MN845763 | 2011 | China | CVA6 |
| JQ964234 | 2011 | China | CVA6 |
| MK106196 | 2013 | China | CVA6 |
| MF285625 | 2011 | China | CVA6 |
| MN845762 | 2011 | China | CVA6 |
| MK106210 | 2011 | China | CVA6 |
| MK106208 | 2011 | China | CVA6 |
| LC126149 | 2009 | Japan | CVA6 |
| LC421552 | 2005 | Japan | CVA6 |
| MF285621 | 2010 | China | CVA6 |
| MK106209 | 2011 | China | CVA6 |
| MK106211 | 2011 | China | CVA6 |
| MH111056 | 2017 | Australia | CVA8 |
| MT814406 | 2014 | France | CVA6 |
| MT814615 | 2018 | France | CVA6 |
| MT350222 | 2014 | China | CVA4 |
| MH086030 | 2015 | China | CVA4 |
| MH086031 | 2014 | China | CVA4 |
| MK391074 | 2016 | China | CVA4 |
| MK967650 | 2018 | China | CVA6 |
| MK106205 | 2015 | China | CVA6 |
| MN845823 | 2017 | China | CVA6 |
| MH086215 | 2015 | China | CVA6 |
| MF285647 | 2014 | China | CVA6 |
| MH086209 | 2015 | China | CVA6 |
| MN845883 | 2017 | China | CVA6 |
| MH086213 | 2015 | China | CVA6 |
| MH716144 | 2015 | China | CVA6 |
| LC506452 | 2016 | VietNam | CVA6 |
| MF578324 | 2014 | VietNam | CVA6 |
| MH086159 | 2015 | China | CVA6 |
| MF285650 | 2014 | China | CVA6 |
| MN845767 | 2014 | China | CVA6 |
| MT814521 | 2014 | France | CVA6 |
| KX064307 | 2015 | China | CVA6 |
| KX064308 | 2015 | China | CVA6 |
| MN845784 | 2016 | China | CVA6 |
| MH086168 | 2015 | China | CVA6 |
| MH086203 | 2015 | China | CVA6 |
| MT814442 | 2014 | France | CVA6 |
| MT350226 | 2016 | China | CVA6 |
| MN845805 | 2017 | China | CVA6 |
| MN845830 | 2017 | China | CVA6 |
| MN845780 | 2016 | China | CVA6 |
| MF285684 | 2016 | China | CVA6 |
| MF285670 | 2015 | China | CVA6 |
| OL839947 | 2018 | China | CVA6 |
| MT814411 | 2018 | France | CVA6 |
| OL839944 | 2018 | China | CVA6 |
| MN845778 | 2015 | China | CVA6 |
| OL839935 | 2018 | China | CVA6 |
| MN845794 | 2016 | China | CVA6 |
| MN845876 | 2017 | China | CVA6 |
| MN845813 | 2017 | China | CVA6 |
| MH536772.2 | 2017 | China | CVA6 |
| MN845844 | 2018 | China | CVA6 |
| MN845810 | 2018 | China | CVA6 |
| MN845877 | 2018 | China | CVA6 |
| OL519577 | 2018 | China | CVA6 |
| MZ491032 | 2019 | China | CVA6 |
| MN845869 | 2018 | China | CVA6 |
| MF285664 | 2015 | China | CVA6 |
| MF285634 | 2013 | China | CVA6 |
| MH086180 | 2015 | China | CVA6 |
| MH086200 | 2015 | China | CVA6 |
| MH086156 | 2016 | China | CVA6 |
| MT350225 | 2017 | China | CVA6 |
| OQ215746 | 2022 | China | CVA6 |
| MF578341 | 2013 | VietNam | CVA6 |
| MF578356 | 2014 | VietNam | CVA6 |
| MF578315 | 2014 | VietNam | CVA6 |
| MF578291 | 2014 | VietNam | CVA6 |
| MF578312 | 2014 | VietNam | CVA6 |
| MF578303 | 2014 | VietNam | CVA6 |
| MF578321 | 2014 | VietNam | CVA6 |
| MF578287 | 2014 | VietNam | CVA6 |
| MF578327 | 2012 | VietNam | CVA6 |
| MF578352 | 2012 | VietNam | CVA6 |
| LC421563 | 2010 | Japan | CVA6 |
| LC421559 | 2010 | Japan | CVA6 |
| KJ541169 | 2012 | China | CVA6 |
| MT814493 | 2014 | France | CVA6 |
| MT814428 | 2014 | France | CVA6 |
| MT814471 | 2014 | France | CVA6 |
| MT814481 | 2014 | France | CVA6 |
| MF373605 | 2010 | China | CVA6 |
| KX189183 | 2011 | China | CVA6 |
| KX189191 | 2013 | China | CVA6 |
| MN845764 | 2012 | China | CVA6 |
| MK106197 | 2013 | China | CVA6 |
| MT814503 | 2010 | France | CVA6 |
| KM114057 | 2008 | Finland | CVA6 |
| MK106195 | 2013 | China | CVA6 |
| LC126156 | 2011 | Japan | CVA6 |
| MT814508 | 2012 | France | CVA6 |
| MT814467 | 2012 | France | CVA6 |
| MT814505 | 2012 | France | CVA6 |
| MT814447 | 2014 | France | CVA6 |
| MT814452 | 2014 | France | CVA6 |
| MT814451 | 2012 | France | CVA6 |
| MT814446 | 2014 | France | CVA6 |
| MT814475 | 2014 | France | CVA6 |
| LC126160 | 2013 | Japan | CVA6 |
| LC126164 | 2013 | Japan | CVA6 |
| KX595286 | 2014 | China | CVA6 |
| MT814484 | 2014 | France | CVA6 |
| MF285673 | 2016 | China | CVA6 |
| MT814458 | 2014 | France | CVA6 |
| MH111035 | 2016 | Australia | CVA6 |
| MT814404 | 2017 | France | CVA6 |
| LC421577 | 2017 | Japan | CVA6 |
| LC421572 | 2015 | Japan | CVA6 |
| MF285675 | 2016 | China | CVA6 |
| LC421575 | 2015 | Japan | CVA6 |
| MT814460 | 2014 | France | CVA6 |
| MH111051 | 2017 | Australia | CVA6 |
| MH111044 | 2016 | Australia | CVA6 |
| MT814410 | 2017 | France | CVA6 |
| MT814424 | 2018 | France | CVA6 |
| ON191507 | 2016 | Senegal | EV-A71 |
| LT719068 | 2003 | CentralAfricanRepublic | EV-A71 |
| MF678310 | 2010 | Australia | CVA2 |
| MG367600 | 2016 | Denmark | EV-A71 |
| LT719044 | 2011 | Madagascar | CVA4 |
| ON191511 | 2019 | Senegal | EV-A71 |
| MG013988 | 2015 | Senegal | EV-A71 |
| ON191512 | 2019 | Senegal | EV-A71 |
| OP255989 | 2018 | Netherlands | CVA5 |
| MH111031 | 2016 | Australia | CVA5 |
| KU761262 | 2015 | Hungary | CVA5 |
| MT081377 | 2014 | USA | CVA5 |
| MF422550 | 2008 | Taiwan | CVA5 |
| KP289364 | 2013 | China | CVA5 |
| HQ728261 | 2009 | China | CVA5 |
| MF422551 | 2008 | Taiwan | CVA5 |
| KP289362 | 2013 | China | CVA5 |
| KP289363 | 2013 | China | CVA5 |
| ON409952 | 2019 | China | CVA5 |
| MH111030 | 2016 | Australia | CVA5 |
| LR798440 | 2017 | Madagascar | EV-A71 |
| LT719062 | 2011 | Madagascar | CVA14 |
| LT719061 | 2007 | Madagascar | CVA14 |
| LT719048 | 2011 | Madagascar | CVA6 |
| LT719047 | 2011 | Madagascar | CVA6 |
| OK570261 | 2011 | Madagascar | EVA120 |
| LT719065 | 2004 | Madagascar | EV-A71 |
| LT719051 | 2011 | Madagascar | CVA7 |
| LT719052 | 2011 | Madagascar | CVA7 |
| LT719064 | 2010 | Madagascar | EV-A71 |
| LT719063 | 2011 | Madagascar | EV-A71 |
| LT719054 | 2011 | Madagascar | CVA7 |
| LT719055 | 2011 | Madagascar | CVA7 |
| LT719053 | 2011 | Madagascar | CVA7 |
| KY888026 | 2016 | USA | EV-A71 |
| MG367595 | 2014 | Denmark | EV-A71 |
| LT617110 | 2012 | France | CVA16 |
| GU942820 | 1958 | Canada | CVA7 |
| LT617109 | 2012 | France | CVA16 |
| LT617107 | 2012 | France | CVA16 |
| LT617115 | 2014 | France | CVA16 |
| LT617106 | 2012 | France | CVA16 |
| LT617108 | 2012 | France | CVA16 |
| GU942823 | 1949 | USA | CVA7 |
| KF501389 | 2010 | China | EV-A71 |
| OQ791544 | 2011 | USA | CVA16 |
| LT617092 | 2003 | Germany | CVA16 |
| OR437337 | 2018 | India | CVA16 |
| LT617104 | 2011 | France | CVA16 |
| MH111068 | 2017 | Australia | CVA16 |
| OR437335 | 2013 | India | CVA16 |
| MT212029 | 2017 | China | CVA16 |
| OR437334 | 2013 | India | CVA16 |
| OR437338 | 2022 | India | CVA16 |
| OP562170 | 1998 | Taiwan | CVA16 |
| JQ746665 | 1997 | Malaysia | CVA16 |
| JQ746667 | 2000 | Malaysia | CVA16 |
| LC506516 | 2004 | Japan | EV-A71 |
| JQ746677 | 2005 | Malaysia | CVA16 |
| OP562200 | 2015 | Taiwan | CVA16 |
| MH010205 | 2017 | China | CVA16 |
| LT617097 | 2010 | Germany | CVA16 |
| OQ791556 | 2014 | USA | CVA16 |
| JQ746671 | 2007 | Malaysia | CVA16 |
| LC506456 | 2008 | VietNam | CVA16 |
| KF055242 | 2010 | China | CVA16 |
| KX595291 | 2014 | China | CVA16 |
| KY425536 | 2014 | China | CVA16 |
| JQ316639 | 2011 | China | CVA16 |
| MZ671842 | 2010 | China | CVA16 |
| MZ671871 | 2014 | China | CVA16 |
| KC755234 | 2010 | China | CVA16 |
| KP266573 | 2008 | China | CVA16 |
| JX068829 | 2010 | China | CVA16 |
| JQ354992 | 2009 | China | CVA16 |
| KY425537 | 2009 | China | CVA16 |
| KY425532 | 2009 | China | CVA16 |
| KC755235 | 2010 | China | CVA16 |
| LT617093 | 2008 | Hungary | CVA16 |
| OQ791540 | 2011 | USA | CVA16 |
| KX372335 | 2011 | Thailand | CVA16 |
| JF738004 | 2010 | Thailand | CVA16 |
| KX372338 | 2012 | Thailand | CVA16 |
| OP562183 | 2010 | Taiwan | CVA16 |
| KX372333 | 2011 | Thailand | CVA16 |
| JF738003 | 2010 | Thailand | CVA16 |
| MT663411 | 2019 | China | CVA16 |
| OM417120 | 2020 | Thailand | CVA16 |
| OP562171 | 1999 | Taiwan | CVA16 |
| FJ198212 | 2008 | China | CVA16 |
| LT617096 | 2009 | Germany | CVA16 |
| MG450666 | 2008 | China | CVA16 |
| KC117317 | 2009 | China | CVA16 |
| JX986742 | 2011 | China | CVA16 |
| KF193629 | 2010 | China | CVA16 |
| LT617098 | 2010 | France | CVA16 |
| LC506455 | 2008 | VietNam | CVA16 |
| LC506515 | 2007 | Japan | EV-A71 |
| KX372336 | 2012 | Thailand | CVA16 |
| MZ671848 | 2011 | China | CVA16 |
| MZ671853 | 2012 | China | CVA16 |
| KY425540 | 2015 | China | CVA16 |
| MZ671854 | 2012 | China | CVA16 |
| KX595295 | 2014 | China | CVA16 |
| KX595293 | 2014 | China | CVA16 |
| KX595292 | 2014 | China | CVA16 |
| MT212022 | 2018 | China | CVA16 |
| MZ671884 | 2015 | China | CVA16 |
| MT211992 | 2018 | China | CVA16 |
| MT212030 | 2018 | China | CVA16 |
| MT212006 | 2016 | China | CVA16 |
| MZ671906 | 2018 | China | CVA16 |
| MT211989 | 2018 | China | CVA16 |
| MZ671881 | 2015 | China | CVA16 |
| MH111067 | 2016 | Australia | CVA16 |
| MZ671905 | 2018 | China | CVA16 |
| MZ671872 | 2014 | China | CVA16 |
| MT211988 | 2017 | China | CVA16 |
| MT212014 | 2017 | China | CVA16 |
| MZ671910 | 2019 | China | CVA16 |
| MT212018 | 2018 | China | CVA16 |
| MZ671898 | 2017 | China | CVA16 |
| MT212035 | 2017 | China | CVA16 |
| MZ671903 | 2018 | China | CVA16 |
| MT179786 | 2007 | China | CVA7 |
| KM609475 | 2012 | China | CVA8 |
| KP289435 | 2013 | China | CVA8 |
| KM609478 | 2012 | China | CVA8 |
| MT648783 | 2015 | China | CVA8 |
| KP036482 | 2012 | China | CVA14 |
| OM638431 | 2019 | China | CVA12 |
| MW079817 | 2017 | China | CVA5 |
| MH086048 | 2016 | China | CVA4 |
| OQ633098 | 2018 | China | CVA4 |
| OL519574 | 2018 | China | CVA4 |
| ON730851 | 2019 | China | CVA4 |
| MH086049 | 2016 | China | CVA4 |
| MN964078 | 2018 | China | CVA4 |
| MN964079 | 2018 | China | CVA4 |
| ON730875 | 2019 | China | CVA4 |
| OL519576 | 2018 | China | CVA4 |
| OL519578 | 2018 | China | CVA4 |
| MT123346 | 2000 | China | EVA120 |
| KX372322 | 2012 | Thailand | EV-A71 |
| FJ357378 | 2003 | Taiwan | EV-A71 |
| MN053430 | 2006 | Malaysia | EV-A71 |
| MG432108 | 1987 | Singapore | EV-A71 |
| AB575923 | 1983 | Netherlands | EV-A71 |
| AB575917 | 1977 | Netherlands | EV-A71 |
| AB575914 | 1971 | Netherlands | EV-A71 |
| AB575918 | 1978 | Netherlands | EV-A71 |
| FJ357384 | 1986 | Taiwan | EV-A71 |
| FJ357383 | 1986 | Taiwan | EV-A71 |
| KX372308 | 2006 | Thailand | EV-A71 |
| HQ728259 | 2009 | China | CVA2 |
| MW410985 | 2009 | China | CVA4 |
| MK391065 | 2011 | China | CVA4 |
| MF285677 | 2016 | China | CVA6 |
| MF422545 | 2008 | Taiwan | CVA4 |
| MT787222 | 2014 | China | CVA4 |
| MH086032 | 2016 | China | CVA4 |
| MH111023 | 2016 | Australia | CVA4 |
| MK658833 | 2018 | China | CVA4 |
| MZ491029 | 2019 | China | CVA4 |
| MF422544 | 2008 | Taiwan | CVA4 |
| MH780726 | 2010 | HongKong | CVA4 |
| MK391064 | 2010 | China | CVA4 |
| MT787220 | 2014 | China | CVA4 |
| MK391063 | 2010 | China | CVA4 |
| KJ541164 | 2010 | China | CVA4 |
| KJ541163 | 2010 | China | CVA4 |
| KT353722 | 2008 | Taiwan | CVA4 |
| MH780728 | 2014 | HongKong | CVA4 |
| MK391068 | 2013 | China | CVA4 |
| MT787221 | 2013 | China | CVA4 |
| MH086037 | 2014 | China | CVA4 |
| MK391070 | 2014 | China | CVA4 |
| MH086050 | 2016 | China | CVA4 |
| MK391069 | 2014 | China | CVA4 |
| JF799986 | 2009 | China | EV-A71 |
| KP308450 | 2012 | Cambodia | EV-A71 |
| JF738002 | 2009 | Thailand | EV-A71 |
| GQ279369 | 2008 | China | EV-A71 |
| FJ607337 | 2008 | China | EV-A71 |
| KP289419 | 2013 | China | EV-A71 |
| OQ355795 | 2012 | China | EV-A71 |
| OQ355744 | 2018 | China | EV-A71 |
| MH111026 | 2016 | Australia | CVA4 |
| MH111024 | 2016 | Australia | CVA4 |
| MT648786 | 2018 | China | CVA8 |
| MF678334 | 2005 | Australia | CVA2 |
| MF678333 | 2008 | Australia | CVA2 |
| MF678322 | 2008 | Australia | CVA2 |
| KX810065 | 2014 | USA | CVA2 |
| MK697689 | 2013 | Australia | CVA2 |
| OP255992 | 2018 | Netherlands | CVA4 |
| OQ319985 | 2020 | UnitedKingdom | CVA4 |
| MH780731 | 2012 | HongKong | CVA4 |
| MT814408 | 2014 | France | CVA6 |
| MT814552 | 2014 | France | CVA6 |
| OP207970 | 2019 | USA | CVA6 |
| MT814547 | 2018 | France | CVA6 |
| ON755025 | 2011 | China | CVA12 |
| KF422143 | 2011 | China | CVA12 |
| KF422142 | 2009 | China | CVA12 |
| ON755027 | 2010 | China | CVA12 |
| MK977588 | 2015 | China | CVA12 |
| MF990300 | 2016 | Ethiopia | CVA16 |
| MK989714 | 2015 | Kenya | CVA16 |
| OP255972 | 2016 | Netherlands | CVA2 |
| OP255994 | 2018 | Netherlands | CVA2 |
| MH111017 | 2016 | Australia | CVA2 |
| MT081367 | 2013 | TrinidadandTobago | EVA120 |
| KU355876 | 2013 | India | EVA114 |
| MF678338 | 2008 | Australia | CVA2 |
| MT648785 | 2016 | China | CVA8 |
| OP255995 | 2018 | Netherlands | CVA4 |
| MH111020 | 2016 | Australia | CVA4 |
| KY271949 | 2015 | USA | CVA4 |
| MH780756 | 2018 | India | CVA6 |
| MH111047 | 2016 | Australia | CVA6 |
| MT814405 | 2018 | France | CVA6 |
| MH111038 | 2016 | Australia | CVA6 |
| MT814576 | 2017 | France | CVA6 |
| MT814562 | 2017 | France | CVA6 |
| MT814563 | 2018 | France | CVA6 |
| MT814577 | 2017 | France | CVA6 |
| MK106189 | 2012 | China | CVA6 |
| LC421554 | 2006 | Japan | CVA6 |
| MT814506 | 2012 | France | CVA6 |
| MT814443 | 2014 | France | CVA6 |
| MT814450 | 2012 | France | CVA6 |
| MT814504 | 2014 | France | CVA6 |
| MT814476 | 2013 | France | CVA6 |
| MT814449 | 2014 | France | CVA6 |
| MT814455 | 2016 | France | CVA6 |
| MT814456 | 2014 | France | CVA6 |
| MT814459 | 2014 | France | CVA6 |
| MN845852 | 2018 | China | CVA6 |
| MH049745 | 2012 | China | CVA6 |
| MN845761 | 2010 | China | CVA6 |
| KX189190 | 2013 | China | CVA6 |
| MT814470 | 2010 | France | CVA6 |
| AB779616 | 2003 | Japan | CVA6 |
| MT814614 | 2012 | France | CVA6 |
| MT814472 | 2015 | France | CVA6 |
| MH111040 | 2016 | Australia | CVA6 |
| MF578370 | 2015 | VietNam | CVA6 |
| LC506451 | 2015 | VietNam | CVA6 |
| KX064295 | 2015 | China | CVA6 |
| MN845839 | 2017 | China | CVA6 |
| MT814468 | 2015 | France | CVA6 |
| MW399171 | 2015 | China | CVA6 |
| MH086150 | 2015 | China | CVA6 |
| MH111043 | 2016 | Australia | CVA6 |
| MT814423 | 2017 | France | CVA6 |
| MH086212 | 2015 | China | CVA6 |
| MN845881 | 2017 | China | CVA6 |
| MN845798 | 2016 | China | CVA6 |
| MH086208 | 2015 | China | CVA6 |
| MH086204 | 2015 | China | CVA6 |
| MH086205 | 2015 | China | CVA6 |
| MT814441 | 2016 | France | CVA6 |
| MK967652 | 2018 | China | CVA6 |
| MT814616 | 2018 | France | CVA6 |
| MF285682 | 2016 | China | CVA6 |
| MN845795 | 2016 | China | CVA6 |
| MN845871 | 2017 | China | CVA6 |
| MN845806 | 2018 | China | CVA6 |
| MH111053 | 2017 | Australia | CVA6 |
| MZ491031 | 2019 | China | CVA6 |
| OR500230 | 2023 | China | CVA6 |
| MN845873 | 2017 | China | CVA6 |
| MT814422 | 2018 | France | CVA6 |
| LK021688 | 2011 | Madagascar | EVA120 |
