# Supplementary material for: Genomic epidemiology of CVA10 in Guangdong, China, 2013–2021
Source: Virol J. 2024 May 30;21:122. doi: 10.1186/s12985-024-02389-9 (PMC11140982; doi:10.1186/s12985-024-02389-9)
Supplement: Supplementary file 7 — Supplementary Material 7 [file 12985_2024_2389_MOESM4_ESM.docx]

**Supplementary Table 6-1** Independent-Sample Mann-Whitney U Test summary for comparison of age distributions

| Statistic | Value |
| --- | --- |
| Total N | 1081 |
| Mann-Whitney U | 110363 |
| Wilcoxon W | 299168 |
| Test Statistic | 110363 |
| Standard Error | 5083.556 |
| Standardized Test Statistic | -6.493 |
| Asymptotic Sig.(2-sided test) | <0.001 |

*The data did not conform to a normal distribution

**Supplementary Table 6-2** Chi-SquareTests summary for comparison of sex distributions

|  | Value | df | Asymptotic Significance (2-sided) | Exact Sig. (2-sided) |
| --- | --- | --- | --- | --- |
| Pearson Chi-Square | 0.225 | 1 | 0.636 |  |
| Continuity Correctionb | 0.169 | 1 | 0.681 |  |
| Likelihood Ratio | 0.224 | 1 | 0.636 |  |
| Fisher's Exact Test |  |  |  | 0.658 |
| Linear-by-Linear Association | 0.224 | 1 | 0.636 |  |
| N of Valid Cases | 1081 |  |  |  |
