# Supplementary material for: Genomic epidemiology of CVA10 in Guangdong, China, 2013–2021
Source: Virol J. 2024 May 30;21:122. doi: 10.1186/s12985-024-02389-9 (PMC11140982; doi:10.1186/s12985-024-02389-9)
Supplement: Supplementary file 8 — Supplementary Material 8 [file 12985_2024_2389_MOESM8_ESM.docx]

**Supplemental Figure 1.** Maximum likelihood trees of CVA10 based on P1 (a), P2 (b) and P3 (c) regions
